# Multiscale Simulation of Phosphofructokinase‑1 Assemblies: Capturing the Interplay between Specific and Transient Interactions

**DOI:** 10.1021/acs.jpcb.5c05346

**Published:** 2025-11-17

**Authors:** Mehrnoosh Khodam Hazrati, Tom Miclot, Stepan Timr

**Affiliations:** J. Heyrovsky Institute of Physical Chemistry, Czech Academy of Sciences, Dolejskova 2155/3, Prague 182 00, Czech Republic

## Abstract

Human phosphofructokinase-1 (PFK1) forms filaments and organizes into large-scale assemblies that are thought to play a key role in the spatial organization of glycolysis. However, the molecular interactions driving this assembly and the isoform-specific tendencies to form such structures remain poorly understood. In this work, we combine coarse-grained and all-atom molecular dynamics simulations to characterize interactions between PFK1 tetramers. Using the Martini and OPEPv7 coarse-grained force fields, we identify key regions mediating transient PFK1–PFK1 interactions and show that these include experimentally identified filament-forming interfaces. At the same time, we find that current coarse-grained models—optimized for nonspecific, transient contacts—lack the resolution to capture the specific side-chain interactions critical for filament stability, as revealed by previous experiments and our all-atom simulations. To address this, we propose enhancing the coarse-grained representation of filament-forming interfaces by introducing additional hydrogen-bonding terms for key residues. This modification improves filament stability and more accurately reproduces the effects of the filament-disrupting Asn-to-Thr mutation. Overall, our work provides a foundation for molecular-level modeling of glycolytic enzyme assemblies and offers a strategy to improve the accuracy of coarse-grained models in capturing the delicate interplay between specific and transient interactions in dynamic protein complexes.

## Graphical abstract

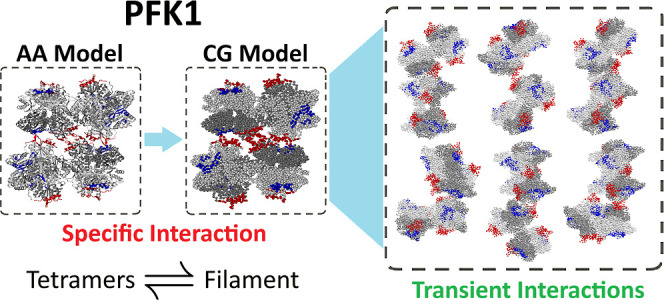

## Introduction

Phosphofructokinase-1 (PFK1), a pivotal enzyme in the glycolytic pathway, catalyzes the conversion of fructose 6-phosphate to fructose 1,6-bisphosphate.^1^ In mammalian cells, three enzyme isoforms carry out this reaction: PFKL (liver), PFKM (muscle), and PFKP (platelet), exhibiting distinct regulatory behaviors.^2,3^ It has been known for several decades that PFKL can form higher-order structures: tetramers of pig PFKL have been found to assemble into long, flexible chains,^4,5^ and large aggregates of rat PFKL have been observed even at low enzyme concentrations.^6^ More recently, the assembly of human PFKL tetramers into large-scale filaments has been reported in vitro.^7^ The ability to form filaments is isoform-specific: while PFKL forms filaments, the PFKP isoform lacks this capability due to differences at the interaction interfaces.^7^

Negative stain electron microscopy (EM) has indicated that human PFKL filaments assemble as stacked tetramers with two distinct interfaces, designated Interface 1 (residues Glu396–Ser401, Gln476–His490, Ala510–Ile519 (Loop 1), and Val693–Asp705 (Loop 2)) and Interface 2 (residues Ala327–Lys356, Gln372–Ser377, and Leu712–Ala718).^7^ High-resolution structures of these stacked tetramers have recently been obtained by cryoEM^8^ (see Figure 1A). Figure 1B shows a PFKL monomer structure in the R-state, highlighting important regions, and Figure 1C displays the relative solvent-accessible surface area (rSASA) per residue, demonstrating highly exposed residues on the protein surface.

**Figure 1:**
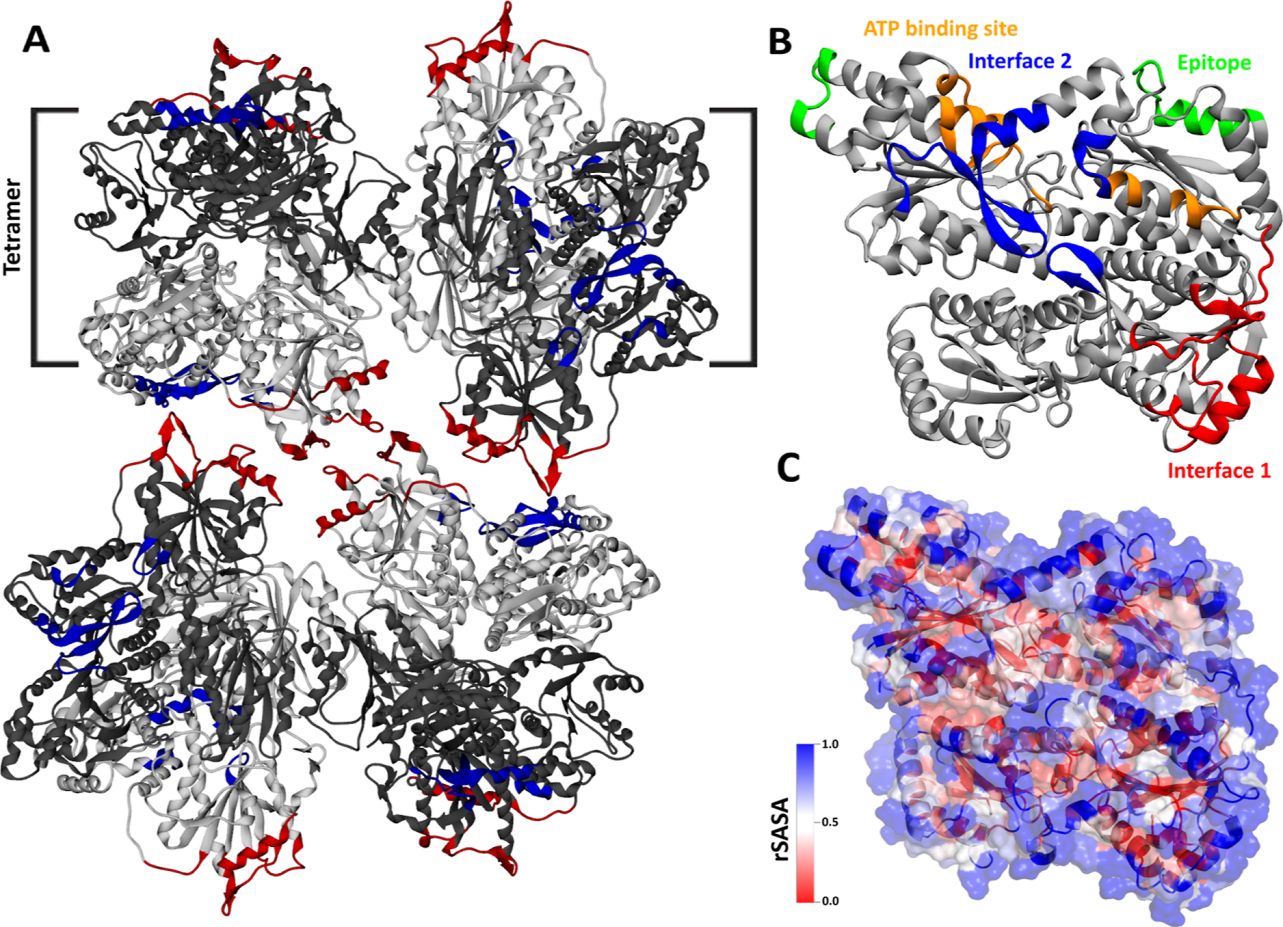
(A) The cryoEM structure of the PFKL filament in the R-state (PDB ID: 8W2I).^8^ Each monomer is colored in gray or silver, and Interfaces 1 and 2 are colored in red and blue, respectively. (B) The cryoEM structure of the PFKL monomer in the R-state. Epitope regions and ATP binding sites are colored in green and orange, respectively. (C) The R-state PFKL monomer is colored based on the relative solvent-accessible surface area (rSASA) values per residue relative to the value for a fully exposed residue. The SASA values corresponding to fully exposed residues are taken from the paper by Tien et al.^9^

Despite the wealth of knowledge concerning the regulation and biochemistry of glycolytic enzymes, our understanding of the mechanisms governing their spatial arrangement within the cell is limited. Besides possible allosteric effects on PFKL activity,^8^ filaments formed by PFKL have been suggested to act as initial seeds of a larger enzyme assembly, also incorporating other glycolytic enzymes and allowing spatial localization and regulation of glycolysis.^10^ In fact, recent cross-linking experiments detected interactions of PFKL Interface 2 with several other glycolytic enzymes but also other biomolecules, such as actin and actin-associated proteins.^11^ Thus, a detailed characterization of the physicochemical factors underlying the self-assembly of PFKL and its interaction with other biomolecular constituents of the cell is a key ingredient for a comprehensive description of localized glycolysis in living cells.

Recent findings indicate that PFK1 forms a complex with other glycolytic enzymes at the plasma membrane to enable localized ATP production.^12^ Therefore, specific and transient interactions that promote the formation of glycolytic enzyme complexes may have important implications for metabolic control in both healthy and cancer cells, warranting further investigation.

The dynamic nature of interactions in glycolytic enzyme assemblies calls for insights from molecular simulations. However, owing to the large sizes of PFK1 tetramers, investigating their interactions is beyond the reach of current all-atom molecular dynamics (MD) simulations, and a coarse-grained modeling approach is required. Among the coarse-grained force fields available for protein simulations, the Martini 3 force field provides a versatile and efficient framework that enables simulations of large biomolecular systems over extended time scales.^13^ On the other hand, the OPEPv7 force field has been optimized to capture the dynamics and transient interactions in crowded protein solutions.^14^

Here, we perform coarse-grained MD simulations of human PFK1 tetramers to shed light on the atomistic details of their interactions and higher-order assembly formation. We characterize structural regions that mediate transient interactions between PFK1 tetramers and show that they include filament-forming interfaces. We also evaluate the efficacy of Martini force fields^15–19^ and OPEPv7 ^14,20^ in modeling the assembly of PFKL filaments and in capturing the different filament-forming propensities of the PFKL and PFKP isoforms. Furthermore, identifying the strength of the Interface 1–Interface 1 interaction as a decisive factor in the stability of the filaments, we compute free energy profiles for this interaction and compare them with results obtained using all-atom MD simulations. Based on these mechanistic insights, we discuss the performance and shortcomings of the current coarse-grained models and suggest ways to improve them.

## Results

### Transient Interactions in the Martini 3 Force Field

Using the Martini 3 force field, we performed 15 μs simulations for PFKL and PFKP, initiating from random positions for the tetramers. Figure 2A illustrates a representative cartoon of two PFK1 tetramers stacked in a filament. The average normalized number of contacts for the PFKL and PFKP monomer surfaces, excluding the Interface 1 and 2 regions (M), Interface 1 (I1), and Interface 2 (I2), are shown in Figure 2B and C, respectively. The normalized number of contacts was calculated using the following expression:

1 $$N_{norm}=\frac{N_{contacts}^{\left(group1,group2\right)}}{{SASA}_{group1}\cdot {SASA}_{group2}}$$

where *N*_contacts_^(group1,group2)^ denotes the number of contacts between the two groups of residues and SASA_group1_ and SASA_group2_ represent the solvent-accessible areas of the two groups. Although we did not observe filament formation throughout the simulation, we still detected Interface 1–Interface 1 and Interface 1–Interface 2 interactions (see Figure 2B,C). At the same time, both PFKL and PFKP formed transient contacts of considerable strength. These transient interactions predominantly involved the filament-forming interfaces, as well as several regions which belong to M, including epitope regions and regions near the adenosine triphosphate (ATP) binding sites (see Figure 2D–G for the contact probabilities). Our MD simulations revealed repeated contact formation in these regions for multiple trajectories (see Figures S1–S3). Specifically, in PFKL, the most frequently interacting residues included the charged residues Lys144 and Lys272, Lys395 (in the immediate vicinity of Interface 1), Glu396 and Lys397 (Interface 1), and Arg695 and Lys696 (Interface 1, Loop 2). In PFKP, Arg705 and Arg707 (Interface 1) were the residues that interacted the most frequently. In total, the two tetramers stayed in contact for nearly the entire trajectory, reaching 94.4% of the time for PFKL and 99.6% for PFKP.

**Figure 2:**
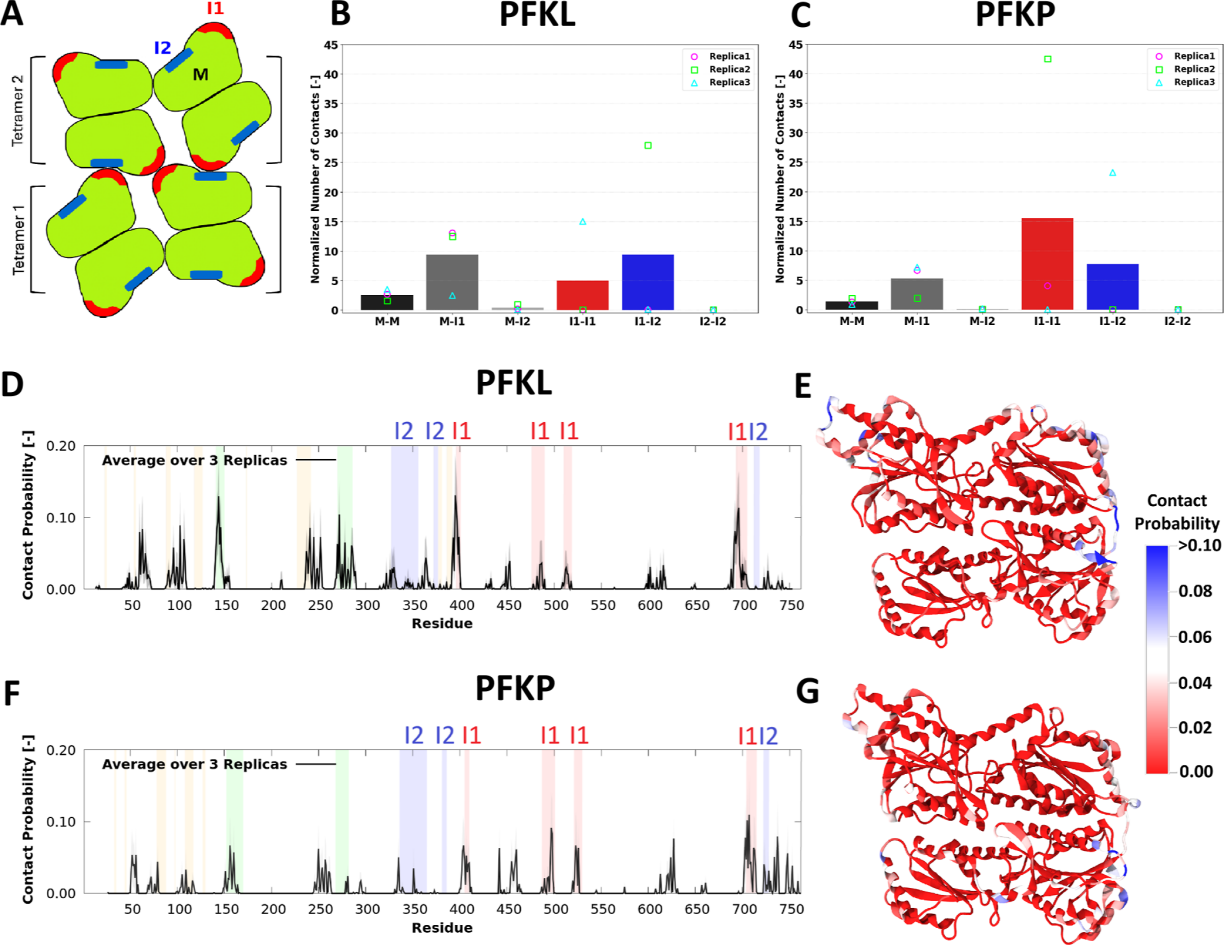
Interactions between PFK1 tetramers in Martini 3 simulations. (A) A representative cartoon of two PFK1 tetramers stacked in a filament. Individual monomers forming each tetramer are shown as a green surface. The red regions show Interface 1, while the blue regions indicate Interface 2 in each monomer. Average number of contacts between the different regions normalized by their SASA values for (B) two PFKL and (C) two PFKP tetramers over 3 simulation replicas started from random initial positions. M indicates the four monomers in each tetramer, excluding the residues of Interfaces 1 and 2. The points indicate the value for each replica. The average contact probability per residue for (D) PFKL and (F) PFKP in simulations started from random initial positions, with the average taken over eight monomers and three replicas. Two beads were considered in contact if their distance was less than 0.7 nm. The shaded areas in gray show the standard error of the mean (SEM). The residue ranges of Interface 1 and Interface 2 are highlighted in red and blue, respectively. The shaded orange and green regions are the ATP binding sites and two important epitope regions, respectively. The above-described residue contact probabilities for (E) PFKL and (G) PFKP, mapped on the structures of the respective monomers. The contact probabilities for each replica are shown separately in Figures S1–S3. Note that since there are only two tetramers in the system, at most two copies of any given residue—each from a different monomer—can be in contact with the other tetramer at a time due to geometric constraints. This imposes a theoretical upper limit of 0.5 on the per-residue average contact probability.

### Comparison of Different Versions of Martini Force Fields in Filament Stability

Further, to investigate the stability of a filament-like arrangement of two PFKL tetramers, we performed a series of 15 μs of coarse-grained simulations started from the cryoEM structure of a preformed PFKL filament.^8^ We compared Martini 3 with two older but widely used versions of the Martini force field, namely, v2.2 ^16–18^ and v2.3P.^19^ To compare the behavior of PFKL with that of PFKP, known to exhibit a low propensity to form filaments,^7^ we repeated the simulations with two PFKP tetramers adopting the same initial positions as the two PFKL tetramers. The results of the coarse-grained simulations and contact analyses for PFKL and PFKP filament stability are presented in Figures S4–S9.

In particular, in the Martini 3 simulations, the Interface 1–Interface 1 interaction site dissociated at approximately 4 μs of coarse-grained simulation time, while the Interface 1–Interface 2 interactions appeared more stable on the given time scale (see Figure S10A). In contrast to PFKL, the PFKP filament structure remained stable on the time scale of the simulations performed using the Martini 3 force field. In addition, Figure S10B,C shows the PFKL filaments during the simulation time for Martini 2 and Martini 2 with polarizable water. Martini 2 and Martini 2 with polarizable water predicted stable filaments for both PFKL and PFKP, indicating a lack of ability in these force fields to distinguish between the PFKL and PFKP isoforms. However, the latter two force fields were previously reported to overstabilize protein–protein interactions.^21–24^

Figure S11A,B shows the short-range Coulomb and Lennard-Jones (LJ) interactions between Interface 1 of two tetramers for PFKL and PFKP, using different Martini force fields. Although Coulombic interactions indicate reduced stability for the PFKP tetramer–tetramer in all Martini force fields compared to those for PFKL, the attraction is dominated by LJ interactions, which fail to capture the observed trend between PFKL and PFKP accurately.

Based on the above results, it is clear that while the significance of filament interfaces (Interfaces 1 and 2) is captured in Martini 3, the model still cannot distinguish between PFKL and PFKP or accurately predict filament formation. This highlights the need to improve the current model of glycolytic enzyme assembly by fine-tuning the coarse-grained force field. A more detailed discussion of this issue is provided in the Discussion section.

### Transient Interactions and Filament Stability in the OPEPv7 Force Field

We further investigated the interactions between PFK1 tetramers using the OPEPv7 force field,^14^ which was optimized to reproduce the dynamics and structure of crowded protein solutions. Figure 3A displays a representative cartoon of two PFK1 tetramers stacked in a filament. The average normalized number of contacts for the PFKL and PFKP monomer surfaces, excluding the Interfaces 1 and 2 regions (M), Interface 1 (I1), and Interface 2 (I2), over 6 simulated replicas (3 replicas with random initial positions and 3 replicas with preformed filament initial structures), in OPEPv7 are presented in Figure 3B,C, respectively. The normalized number of contacts was calculated using the expression (1). Similarly to Martini 3, the two tetramers were found to engage in frequent transient interactions over the simulation time (150 μs), spending 53.6% and 63.9% of the total simulation time in contact for PFKL and PFKP, respectively.

**Figure 3:**
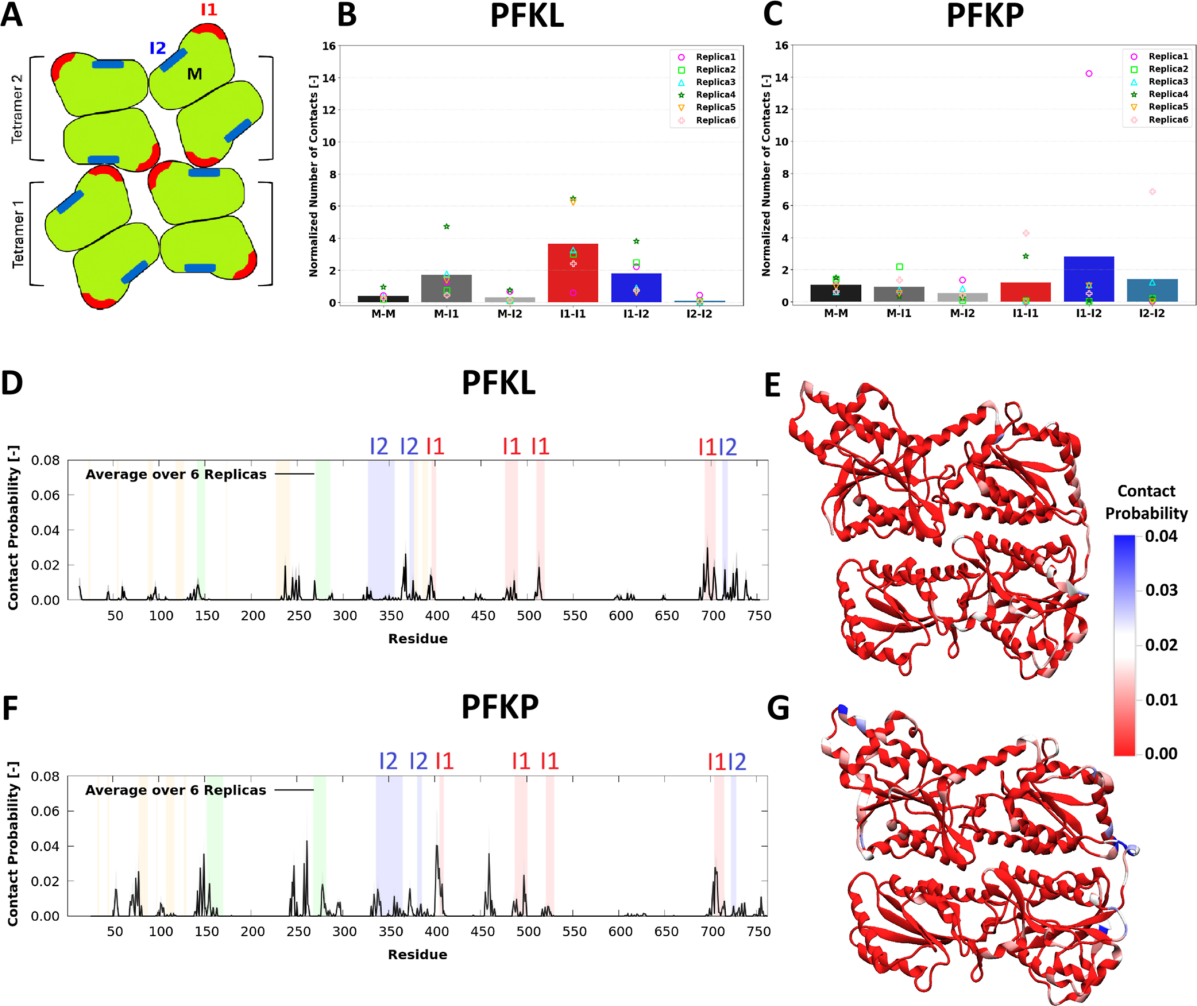
Interactions between PFK1 tetramers in OPEPv7 simulations. (A) A representative cartoon of two PFK1 tetramers stacked in a filament. Individual monomers forming each tetramer are shown as a green surface. The red regions show Interface 1, while the blue regions indicate Interface 2 in each monomer. The average number of contacts between the different regions normalized by their SASA values for (B) two PFKL and (C) two PFKP tetramers over six simulated replicas (three started from random initial positions and three from preformed filament structures). M indicates the four monomers in each tetramer, excluding the residues of Interfaces 1 and 2. The points indicate the value for each replica. The average contact probability per residue for (D) PFKL and (F) PFKP. The values are averaged over the eight monomers and six replicas. Two beads were considered in contact if their distance was less than 0.7 nm. The shaded areas in gray show the standard error of the mean (SEM). The residue ranges of Interface 1 and Interface 2 are highlighted in red and blue, respectively. The shaded orange and green regions are the ATP binding sites and two important epitope regions, respectively. The above-described residue contact probabilities for (E) PFKL and (G) PFKP, mapped on the structures of the respective monomers. The contact probabilities for each replica are shown separately in Figures S12–S14.

The interactions between the tetramers involved residues of the filament-forming interfaces—in particular, those belonging to Interface 1—epitope regions, and regions near ATP-binding sites (see Figure 3D–G). In PFKL, residues in specific regions, including Arg695 and Lys696 from Loop 2 of Interface 1, Glu692 near Loop 2 of Interface 1, Arg513 in Loop 1 of Interface 1, Lys715 from Interface 2, Arg366 and Asp368 near Interface 2, and Lys395 near Interface 1, contributed to the observed interactions. The residues such as Asp237, Glu724, Lys727, and Asp728 also appeared transiently. For PFKP, along with key interactions for residues Arg705–Arg707 at Loop 2 of Interface 1, other transient interactions were observed involving residues Ser142, Leu145, Glu146, Ala149, Glu245, and Thr278.

Importantly, OPEPv7 and Martini 3 agree on the primary regions involved in transient interactions: the filament-forming interfaces, epitope regions, and ATP-binding regions. Both force fields also identify several particularly strongly interacting residues: Lys395, Arg695, and Lys696 in PFKL and Arg705–Arg707 in PFKP. Together, these observations suggest that, beyond their critical role in filament formation, the filament-forming interfaces are also important actors in other, more transient modes of interaction between PFK1 tetramers—and possibly also between PFK1 and other components of the glycolytic metabolon.

The OPEPv7 model, regardless of initial structural configurations, did not produce stable filaments in our simulations. The dynamics of a typical crowded protein solution is mainly governed by weak, transient interactions between proteins rather than the formation of specific, strongly bound complexes.^14^ Our results thus highlight the challenges in capturing both transient and specific protein–protein interactions using a single coarse-grained model.

### Critical Role of Interface 1–Interface 1 Interaction

Besides the heavy involvement of Loop 2 of Interface 1 in transient interactions observed in both Martini 3 and OPEPv7 simulations, our Martini 3 simulations pointed to an important role of the Interface 1–Interface 1 interaction in determining filament stability. The recent cryoEM structures of PFKL filaments^8^ identified Loops 1 and 2 (see Figure 4) as the mediators of the Interface 1–Interface 1 interaction. A sequence comparison between PFKL and PFKP reveals that Loop 2 contains more sequence differences than Loop 1, both in terms of the fraction of mutated residues and the fraction of residues with significantly different physicochemical properties, based on the classification of residues introduced by Pommié et al.^25^ (see Tables S1–S6). Both Loop 2 and, to a lesser extent, Loop 1 are enriched in charged, polar, and hydrophilic residues in PFKP compared to PFKL (see Tables S5 and S6). The higher density of charged residues within these loops in PFKP may promote the formation of transient, nonspecific contacts (see Figures 2 and 3). However, such transient interactions are distinct from the highly specific and complementary interactions required for stable filament assembly. In fact, substituting Asn702 with Thr (N702T)—a single PFKP-like mutation in Loop 2 with no effect on total charge or polarity—was experimentally shown to disrupt filament formation.^8^

To characterize the interacting residue pairs at Interface 1–Interface 1 with all-atom resolution, we considered the binding of two identical PFKL fragments containing Interface 1 (see Figure 4A) and performed three 500 ns replicas of atomistic MD simulations to relax the cryoEM structure. Using the MICLOT tool,^26^ we then identified stable residue pairs and their nonbonding interaction along the trajectories. We found that the Interface 1–Interface 1 interaction was stabilized by the presence of salt bridges and a significant number of hydrogen bonds. As shown in Figure 4B, the backbone of Asn702 forms a hydrogen bond with Gly512 from the opposing fragment, while its side chain forms two hydrogen bonds with the side chain of the other Asn702 and an additional hydrogen bond with Arg511. For a comparison with PFKP Interface 1–Interface 1, where Asn702 is replaced with Thr, see Figure S15. The involvement of Asn702 in the multiple hydrogen bonding interactions in PFKL Interface 1–Interface 1 explains the key role of this residue observed experimentally in stabilizing the filament.^8^ In contrast, the Asn702 residue at Interface 1–Interface 2 is unlikely to contribute significantly to filament stability, as the spatial arrangement of the filament prevents Asn702 from interacting strongly with Interface 2 residues (see Figure S16). Consequently, the destabilizing effect of the N702T mutation is likely confined to the Interface 1–Interface 1 contact. At the coarse-grained level of Martini 3 and OPEPv7, hydrogen-bonding interactions are implicitly included in the orientation-averaged potentials attributed to individual beads. However, it is not a priori clear whether this implicit description is sufficient to stabilize a complex.

**Figure 4:**
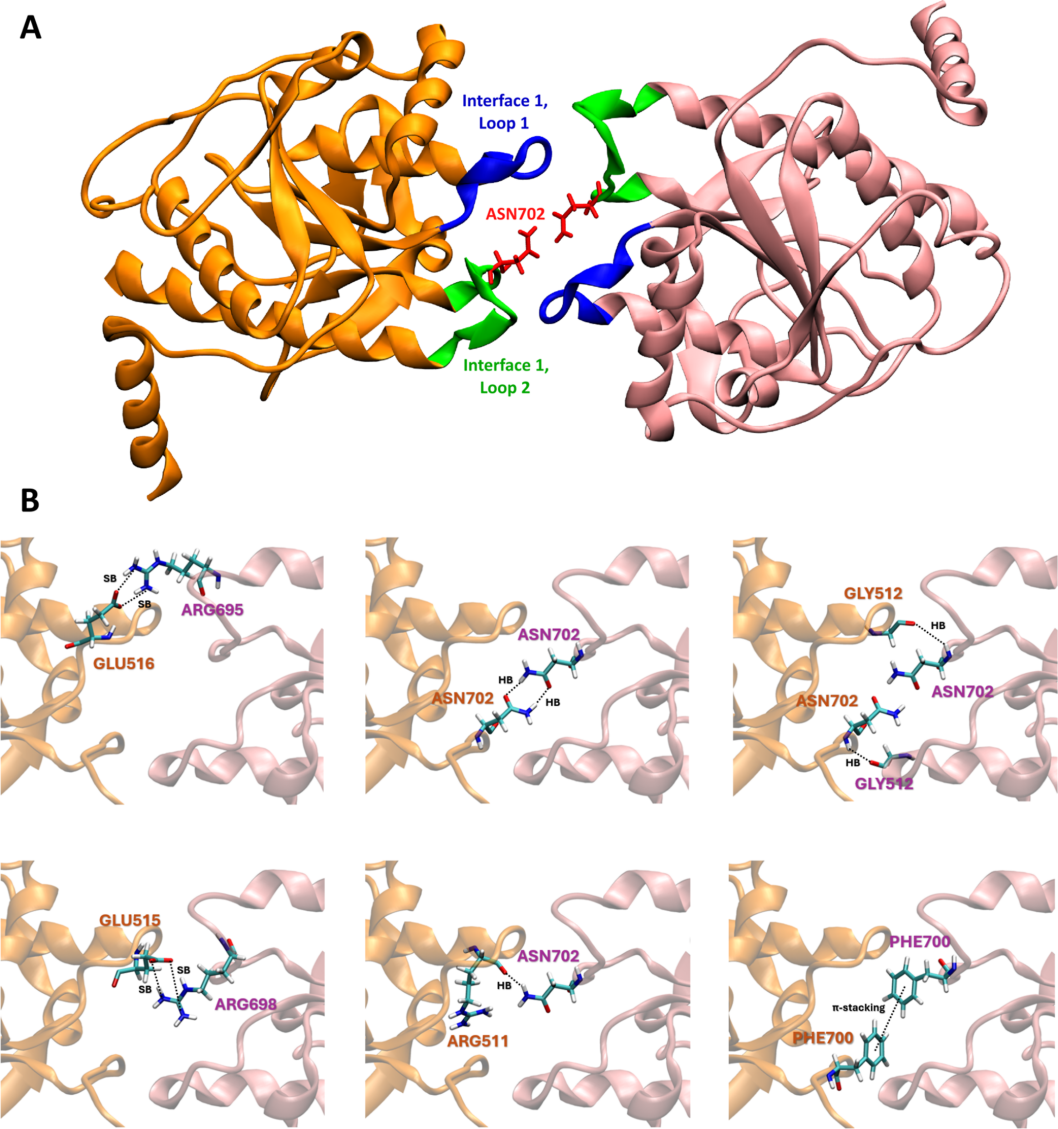
(A) Atomistic representation of the initial structures of the two native PFKL fragments, engaged in Interface 1–Interface 1 interaction, as obtained by cryoEM.^8^ Fragments A and B are shown in orange and pink, and Loop 1 and Loop 2 of Interface 1 are shown in blue and green, respectively. The Asn702 residues of each fragment are highlighted in red. (B) Zoom-in view of the most interacting residues during three 500 ns replicas of atomistic MD simulations performed to relax the cryoEM structure. SB stands for “salt bridge”, and HB stands for “hydrogen bond”.

In PFKL, additional interfacial stability is provided by salt bridges between Glu516–Arg695 and Glu515–Arg698 across fragments, as well as aromatic stacking between Phe700 residues. Dissection of the Interface 1–Interface 1 structure into distinct regions based on residue hydration^27^ using the MICLOT tool^26^ shows that the more buried core and support regions are identical in size, each being formed by approximately 11–12 residues, while only 8–9 residues form the more solvent-exposed rim (see Figure S17), combining its interacting part and its noninteracting surface (NIS) part.^28^ This shows that the more buried regions play an important role in the association of two Interfaces 1. The significance of the support regions, largely buried before protein complex formation, has already been discussed in a previous study.^26^

Furthermore, to investigate the energetics of the Interface 1–Interface 1 interaction, we performed enhanced sampling simulations to estimate the binding free energy at both the atomistic and coarse-grained levels. Binding free energy profiles (Figure 5A,B), obtained from replica-exchange umbrella sampling (REUS)^29^ simulations of two PFKL fragments containing Interface 1, show that all-atom simulations predict a stable Interface 1–Interface 1 interaction for native (wild-type) PFKL while also capturing the substantial difference between native and N702T mutant fragments.

**Figure 5:**
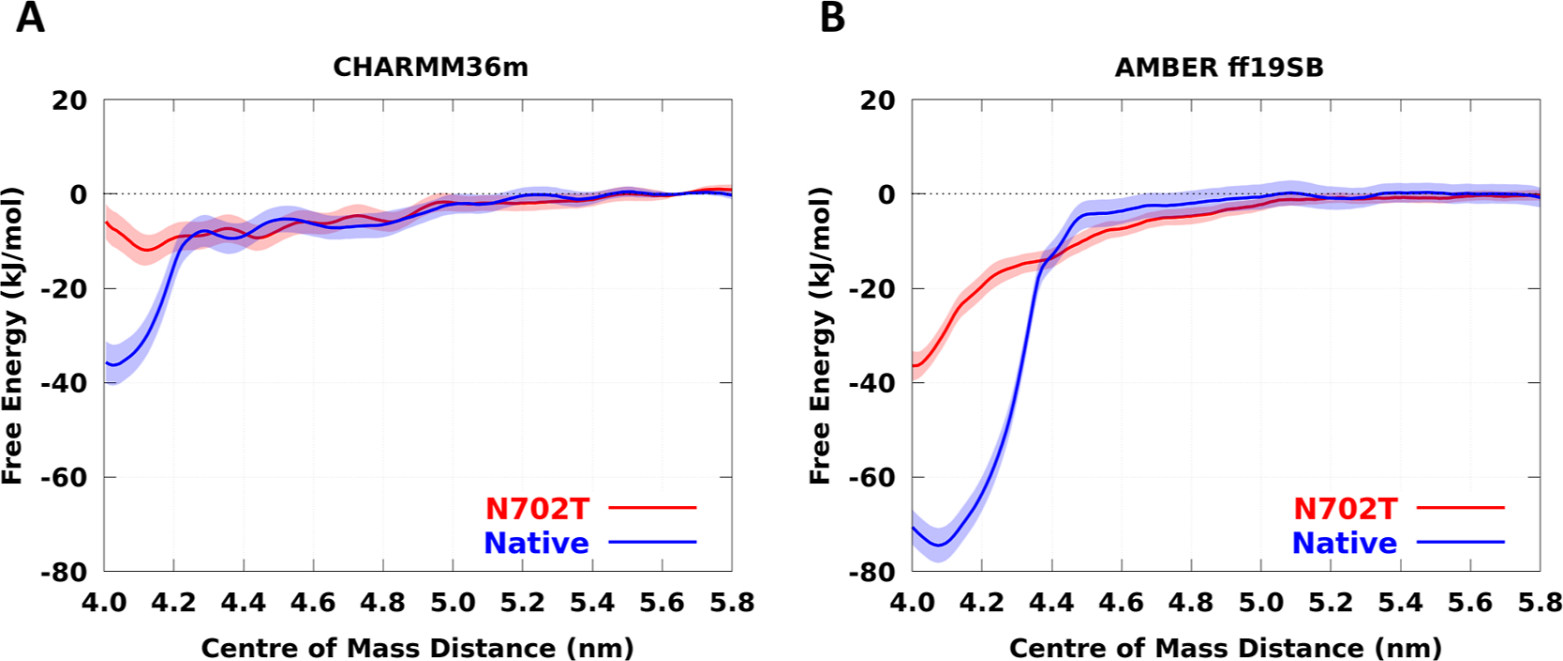
Free energy profiles of Interface 1–Interface 1 interaction for native PFKL and its N702T mutant (a PFKP-like mutation) calculated from all-atom simulations of protein fragments using (A) CHARMM36m and (B) AMBER ff19SB force fields. The free energy difference between PFKL and PFKP estimated from experimental data is about 20 kJ mol^–1^. The shaded areas represent error bars calculated by using a bootstrap method.

Using the CHARMM36m force field,^30^ the binding free energy difference between the native and mutant fragments is approximately 25 kJ mol^–1^, close to the value of 21 kJ mol^–1^ estimated from mass photometry data published by Lynch et al.^8^ (see the Supporting Information for details of the calculation). The AMBER ff19SB force field,^31^ predicts a larger energy difference of around 40 kJ mol^–1^.

In contrast, as Figure 6 shows, the Martini 3 coarse-grained force field yields a marginally stable Interface 1–Interface 1 interaction, displaying a negligible energy difference between the native and mutant fragments, thus failing to capture the significant destabilization observed experimentally. In the case of OPEPv7, the tetramers detached early in the simulations, indicating very weak binding affinity and thus not warranting further exploration using enhanced sampling simulations.

## Discussion

The PFK1 organization in the cell is believed to fulfill a regulatory role in metabolism that extends beyond its well-established catalytic function.^7,33^ The formation of higher-order assemblies may facilitate localized control of glycolytic flux, enhance substrate channeling, or enable the spatial organization of metabolic pathways in response to cellular needs.^7,34,35^ Furthermore, the dynamic and reversible nature of these assemblies supports the hypothesis that PFK1 may also fulfill moonlighting roles, such as participating in signaling pathways or acting as a scaffold for other proteins.^36^ Molecular dynamics simulations are beginning to enable a more comprehensive understanding of allosteric transitions in PFK1, offering spatial and temporal resolution that was previously unattainable.^8^

The aim of this work was to characterize interactions underlying PFK1 assembly formation using molecular dynamics simulations. To achieve our goal, we investigated the interactions between two tetramers of PFKL or PFKP, employing the Martini and OPEPv7 coarse-grained force fields, the latter being optimized to capture the dynamics and transient contacts in crowded protein solutions.^14^ To further validate our study and gain deeper insight into the mechanism of PFKL filament formation, we performed replica-exchange umbrella sampling (REUS)^29^ simulations on PFKL Interface 1 fragments and their N702T mutant and compared the coarse-grained free energy profiles with those obtained with detailed all-atom models.

Our simulations using the Martini 3 force field and the OPEPv7 model show that the filament-forming interfaces, particularly Interface 1, are involved in transient contacts between PFK1 tetramers even when no filaments are formed. These contacts, which also involve other regions of the protein surface, including epitope regions and residues surrounding ATP-binding sites, may influence PFK1 activity and allosteric regulation inside glycolytic enzyme assemblies. It has been suggested that, by facilitating substrate channeling or allosteric regulation, these higher-order complexes may enhance the efficiency and coordination of glycolysis within the cell.

As confirmed by our all-atom simulations, the stability of the filament structure relies on a detailed arrangement of side chains and their specific interactions. Correctly describing these critical interactions while avoiding the overstabilization of nonspecific transient contacts remains challenging for current coarse-grained models. Our results show that improvements of existing coarse-grained force fields are necessary to capture both filament stability and the difference between the two PFK1 isoforms.

Therefore, to correctly describe PFK1 filament assembly, the current coarse-grained force fields need to be modified to account for the specific hydrogen-bonding interactions that are key to the distinct behavior of the PFKL and PFKP isoforms. A promising avenue is opened up by the OLIVES model,^32^ a recently introduced addition to the Martini 3 force field. The OLIVES model is a Go̅-like model^37^ that serves to stabilize protein structure via the formation of hydrogen bonds and native contacts within the Martini 3 coarse-grained force field.^32^ However, we found that the current Martini 3 force field in combination with the standard OLIVES model yielded an overestimation in the binding free energies of the native PFKL fragments and the N702T mutant and was not able to correctly predict the trend between them (see Figure S18).

As a minimal, localized modification informed by physical insight into the role of the buried Asn702–Asn702 pair, we added an extra hydrogen-bonding term to the Asn702 residue in the Martini 3 force field to improve the stability of the complex and correctly capture the destabilizing effect of the N702T mutation. The modification is supported by both experimental evidence^8^ and our atomistic simulations and is further justified by our detailed atomistic-level interaction analysis. Specifically, we described the hydrogen-bonding interaction between the two Asn702 side chains using the corresponding OLIVES^32^ intermolecular parameter (see Methods), based on a realistic hydrogen bond energy derived from ab initio calculations^38^ and consistent with the interaction strength of a partially desolvated bidentate Asn–Asn pair.^39^

The modified force field correctly predicted the native Interface 1–Interface 1 complex (see Figure S19), resulted in a free energy value more consistent with the atomistic range, and correctly differentiated between native and mutant fragments (see Figure 6).

**Figure 6:**
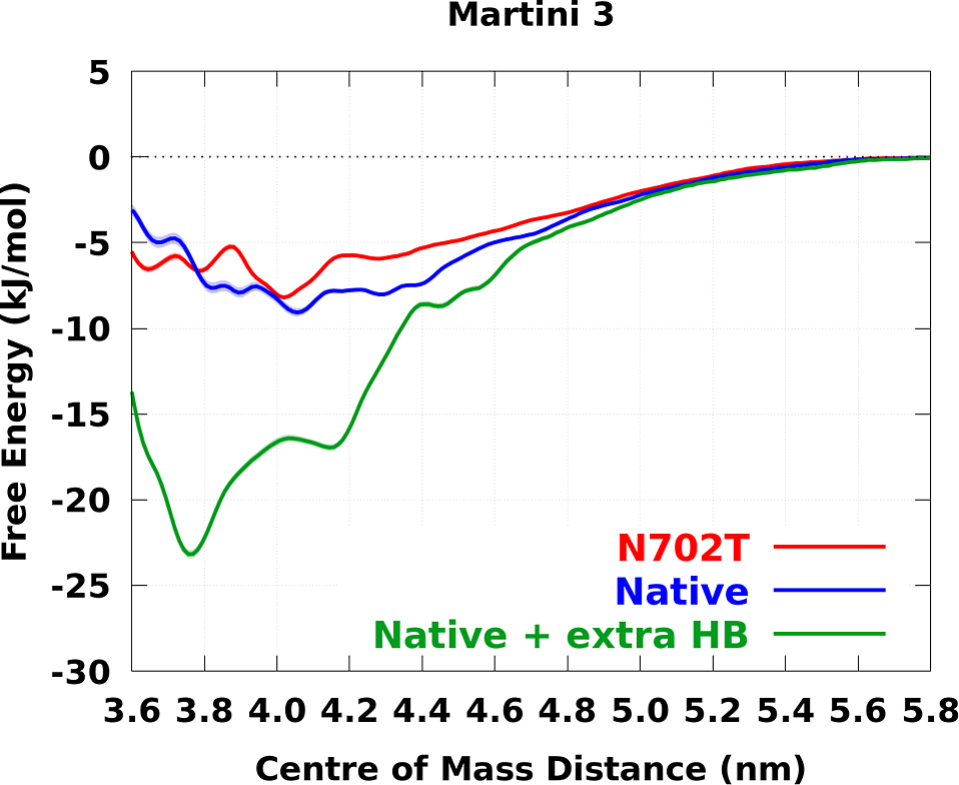
Free energy profiles of Interface 1–Interface 1 interaction for native PFKL and its N702T mutant (a PFKP-like mutation) obtained using Martini 3 and Martini 3 with an extra hydrogen bonding (HB) term in which the OLIVES^32^ quaternary bond parameter for Asn702–Asn702 side chains is used. The shaded areas represent error bars calculated by using a bootstrap method.

To further evaluate our modified Martini 3 force field, which includes an additional hydrogen-bonding term for the side chains of Asn702 residues, we performed 10 μs unbiased simulations (three replicas) of two PFKL tetramers randomly placed in the simulation box. Although filament formation was not observed within the limited simulation time scale, the extra hydrogen-bonding term significantly strengthened the Interface 1–Interface 1 interactions (see Figure S20), a prerequisite for filament formation.

While a minimal adjustment of the LJ parameters for the Asn702 side chain already leads to a marked improvement in the free energy profile, further optimization involving additional residues could enhance the agreement even more. Table S7 compares the all-atom and coarse-grained residues in the PFKL Interface 1–Interface 1 complex (Loop 1 and Loop 2), highlighting potential candidates for such further modifications.

Our results indicate that future improvements in the models of specific soluble protein–protein complexes should be directed toward addressing the limitations of current coarse-grained models. However, at present, the existing Martini 3 and OPEPv7 models necessitate the application of biases, such as adding extra hydrogen-bonding terms to the quaternary network, in order to improve the representation of specific soluble complexes.

## Conclusions

This work addresses the challenges of accurately modeling PFK1 assembly formation and isoform-specific differences by combining coarse-grained and all-atom simulations. All-atom simulations reveal that the stability of the PFKL filament relies heavily on precise side-chain arrangements and Interface 1–Interface 1 interactions. While existing coarse-grained models struggle to capture these specific interactions, Martini 3 with hydrogen bond modification demonstrates promising performance. Our modified Martini 3 model effectively stabilizes the PFKL filament, enabling discrimination between the native form and the PFKP-like Asn702 to Thr mutant. Importantly, both the Martini 3 and OPEPv7 coarse-grained models predict a significant involvement of the filament-forming interfaces—particularly Interface 1—in mediating transient contacts between PFK1 tetramers. This finding suggests that the interfaces are not only critical for filament stabilization but also contribute to dynamic regulatory interactions between tetramers in the crowded cytoplasmic environment. Such transient contacts may influence the higher-order organization of PFK1 and its incorporation into the glycolytic metabolon. Characterizing these interactions at both the coarse-grained and atomistic levels provides valuable insight into the balance between assembly stability and functional flexibility and highlights the broader significance of filament interfaces in the dynamic behavior of PFK1 assemblies.

## Methods

### Structure Preparation

We used the R-state human liver phosphofructokinase (PFKL) filament structure, which is now available in the PDB database under the PDB ID: 8W2I.^8^ We also used this structure as a reference to assemble an R-state human platelet phosphofructokinase (PFKP) filament from tetramers (PDB ID: 4XZ2).^55^

To make the PFKL fragment, the 237 residues in close proximity to PFKL Interface 1 (Gly375-Gly540 and Phe670-Trp740) were selected. To create a PFKP-like fragment, the asparagine (Asn) residue at position 702 in the PFKL fragment was mutated to a threonine (Thr) residue. This mutant fragment is a PFKP-like mutation.

All the representations of structures were created using VMD version 1.9.3.^40^

### Coarse-Grained Simulations with Martini Force Fields

Martinize2 ^41^ was used to convert atomic structures into coarse-grained Martini structures and topologies. The coarse-grained complexes of PFKL and PFKP tetramers were simulated using the GROMACS version 2022.5 software package^42,43^ and coarse-grained Martini 3 (v3.0.0),^15^ Martini 2 (v2.2),^16–18^ and Martini 2 with polarized water (v2.3P)^19^ force fields. Elastic networks (ENs) were employed on the backbone beads for each tetramer with a 700 kJ mol^–1^ nm^–2^ harmonic force constant.

After solvation, the system was supplemented with Na^+^ and Cl^–^ ions at physiological concentrations of 150 mM. Additional Cl^–^ ions were added to balance the net charge. The systems were equilibrated through several short NVT steps, followed by 500 ns of NPT equilibration. Reaction field electrostatics with a cutoff of 1.2 nm and ϵ_r_ = 15 were applied for Martini 3 and Martini 2. Van der Waals interactions were calculated using the potential shift with the Verlet cutoff-scheme method and a cutoff of 1.2 nm. In the case of Martini 2 with polarized water systems, ϵ_r_ = 2.5 was used, van der Waals interactions were calculated using the force-switch method and a cutoff of 1.2 nm, and the Particle Mesh Ewald (PME) method^44^ was applied to the long-range electrostatic interactions. Temperature and pressure were kept constant at 300 K and 1 bar using the v-rescaling thermostat^45^ and the c-rescale barostat^46^ with a coupling constant of 1 and 12 ps, respectively. The Parrinello–Rahman barostat^47^ was used for production runs with a coupling constant of 12 ps and compressibility of 3 × 10^–4^ bar^–1^. The integration time step was 20 fs, and periodic boundary conditions were applied in all directions. Production molecular dynamics (MD) runs were conducted for 15 μs with three replicas for each system.

We started the simulations from preformed filament configurations. However, in Martini 3, we simulated a total of six replicas, with the two tetramers in the simulation box being placed randomly for the other three replicas. The box sizes were roughly 25 × 25 × 18 nm^3^, while for the randomly positioned tetramers in Martini 3, we increased the box size to 28 × 28 × 20 nm^3^.

### Coarse-Grained Simulations with the OPEPv7 Force Field

The PFKL and PFKP complexes were subjected to further investigation utilizing the OPEPv7 elastic network model,^14,20^ as implemented in the OpenMM version 8.1 simulation package.^48^ The OPEPv7 force field represents the side chains of protein residues as single interacting beads with chemical specificity. The PFKL and PFKP structures were maintained with the assistance of EN interconnecting backbone and side-chain beads, with the nonbonded cutoff distance set to 1.0 nm. The Langevin integrator was used to simulate the dynamics of the systems. The temperature of the heat bath was maintained at 298 K. The friction coefficient, γ, which determines how strongly the system is coupled to the heat bath, was set to 10 ps^–1^ for both PFKL and PFKP. A cubic box with dimensions of 25 nm was used as the simulation box. A total of 50,000 minimization steps were performed, followed by an MD production run of 150 μs for each system. The time step size was 20 fs. A total of six replicas were simulated. For three replicas, we started from preformed filament configurations, but for the other three replicas, we randomly placed the two tetramers in the simulation box.

### Atomistic Unbiased MD Simulations of PFKL Fragments

Two PFKL fragments were placed in a simulation box of the size of 13 × 13 × 10 nm^3^, while their Interface 1 was in contact. A flat-bottom-high potential was applied to the residues Val722-Asp728 of each fragment to avoid unrealistic interactions between the two fragments, sterically impossible in the filament. The CHARMM36m force field^30^ and the TIP3P water model^49^ were employed to generate the configuration and topology files. K^+^ and Cl^–^ ions were added to the system at physiological concentrations of 150 mM, and extra Cl^–^ was used to balance the net charge in the system. After minimization, the systems were equilibrated in several NVT and NPT runs with positional restraints on the protein backbones that were gradually loosened. Temperature (300 K) and pressure (1 bar) were controlled using the Berendsen thermostat^50^ and barostat,^51^ with a coupling constant of 1 ps. Short-range electrostatics and van der Waals interactions were truncated and shifted to zero at 1.2 nm, while long-range electrostatic interactions were treated with the Particle Mesh Ewald (PME) method,^44^ using a grid spacing of 1.2 nm, a real space cutoff of 1.2 nm, and PME order 4. The integration time step was set at 1 fs, and periodic boundary conditions were applied in all directions.

For MD production runs, the temperature was maintained at 300 K using the v-rescale thermostat^45^ with a coupling constant of 0.1 ps. An isotropic pressure of 1 bar was applied using the Parrinello–Rahman algorithm^47^ with a coupling constant of 1.0 ps and compressibility of 4.5 × 10^–5^ bar^–1^. Short-range electrostatics and van der Waals interactions were truncated and shifted to zero at 1.2 nm, while long-range electrostatic interactions were treated with the PME method,^44^ using a grid spacing of 1.2 nm, a real space cutoff of 1.2 nm, and PME order 4. The integration time step, owing to the hydrogen mass repartitioning (HMR) approach used in this set of unbiased simulations, was set to 4 fs. We performed 500 ns of unbiased atomistic MD simulations to relax the cryoEM structures for three replicas of the PFKL fragments. To analyze the interactions between residue pairs at Interface 1, the three MD replicas were divided into 16 frames (a total of 48 frames) and analyzed using MICLOT, our recently developed interaction analysis tool.^26^

### Atomistic Free Energy Calculations of Fragment Interactions

To gain further insight into the nature of the interactions between Interface 1 of the tetramer–tetramer complexes in PFKL and PFKP, we have calculated the free energy profiles of native PFKL and its N702T mutant fragments.

The CHARMM36m force field^30^ and the TIP3P water model^49^ were employed to generate the configuration and topology files. Na^+^ and Cl^–^ ions were added to the system at physiological concentrations of 150 mM, and extra Cl^–^ was used to balance the net charge in the system. The systems were equilibrated under an NVT ensemble with shallow positional restraints on the protein backbones and side chains. Temperature (300 K) was controlled using the Nose–Hoover thermostat^52^ with a coupling constant of 1 ps. Short-range electrostatics and van der Waals interactions were truncated and shifted to zero at 0.9 nm, while long-range electrostatic interactions were treated with the Particle Mesh Ewald (PME) method,^44^ using a grid spacing of 0.9 nm, a real space cutoff of 0.9 nm, and PME order 4. The integration time step was set at 1 fs, and periodic boundary conditions were applied in all directions.

To study fragment–fragment interactions, the center of mass (COM) of each fragment was pulled for 40 ns with a pulling rate of 0.00005 nm ps^–1^ and a harmonic potential force constant of 1000 kJ mol^–1^ nm^–2^. During pulling and replica-exchange umbrella sampling (REUS),^29^ temperature was maintained at 300 K using the v-rescale thermostat^45^ with a coupling constant of 0.1 ps, and isotropic pressure of 1 bar was applied using the Parrinello–Rahman algorithm^47^ with a coupling constant of 1.0 ps and compressibility of 4.5 × 10^–5^ bar^–1^. Short-range electrostatics and van der Waals interactions were truncated and shifted to zero at 1.2 nm, while long-range electrostatic interactions were treated with the PME method,^44^ using a grid spacing of 1.2 nm, a real space cutoff of 1.2 nm, and PME order 4. The integration time step was set at 2 fs. Subsequently, REUS simulations were conducted for 64 windows equally spaced by 0.03 nm for a simulation time of 87 ns.

The simulations were repeated using the AMBER ff19SB force field^31^ and the OPC water model.^53^ Na^+^ and Cl^–^ ions were added to the system at physiological concentrations of 150 mM, and extra Cl^–^ was used to balance the net charge in the system. The systems were equilibrated under an NVT ensemble with positional restraints on the protein backbones and side chains. A Nose–Hoover thermostat^52^ with a coupling constant of 1.0 ps was used to maintain the temperature at 300 K. Van der Waals interactions were truncated at the cutoff distance of 0.9 nm, while long-range electrostatic interactions were treated with the PME method,^44^ using a grid spacing of 0.9 nm, a real space cutoff of 0.9 nm, and PME order 4. Periodic boundary conditions were applied in all directions, and the integration time step was set to 1 fs.

To study fragment–fragment interactions, the center of mass (COM) of each fragment was pulled for 100 ns with a pulling rate of 0.00005 nm ps^–1^ and a harmonic potential force constant of 1000 kJ mol^–1^ nm^–2^. During pulling and REUS, the temperature was maintained at 300 K using the Nose–Hoover thermostat^52^ with a coupling constant of 1.0 ps. The pressure was maintained at 1 bar using the Parrinello–Rahman barostat,^47^ with a coupling constant of 5.0 ps and compressibility of 4.5 × 10^–5^ bar^–1^. The van der Waals interactions were truncated at a cutoff distance of 1.4 nm, and long-range electrostatic interactions were treated with the PME method,^44^ using a grid spacing of 1.4 nm, a real space cutoff of 1.4 nm, and PME order 4. Subsequently, REUS simulations were conducted for 64 windows equally spaced by 0.03 nm for a simulation time of 50 ns for each window.

This window spacing was selected to ensure sufficiently high exchange probabilities (e.g., >0.4). The convergence analysis is reported in the Supporting Information, Figures S21 and S22. In Figure 5, we plotted the free energy profiles obtained from the second halves of the umbrella trajectories. The force constants used for the REUS simulations are listed in Equation S14 in the Supporting Information. The Weighted Histogram Analysis Method (WHAM)^54^ was then used to reconstruct the free energy profiles for the second half of the trajectories.

### Coarse-Grained Free Energy Calculations of Fragment Interactions

All-atom fragment structures were converted to Martini coarse-grained models using Martinize2.^41^ The coarse-grained systems were equilibrated through several short NPT runs, followed by 100 ns of MD runs. For equilibration, the v-rescale thermostat^45^ and c-rescale barostat^46^ were applied, maintaining the temperature at 300 K with a coupling constant of 1 ps and pressure at 1 bar with a coupling constant of 12 ps and compressibility of 3 × 10^–4^ bar^–1^. Reaction field electrostatics with a cutoff of 1.1 nm and ϵ_r_ = 15 were used, and van der Waals interactions were computed via potential-shift with the Verlet cutoff-scheme method with a cutoff of 1.1 nm. The integration time step was 20 fs, and periodic boundary conditions were applied in all directions. The fragment COM pulling was conducted over 350 ns with a pulling rate of 0.000005 nm ps^–1^ and a harmonic potential force constant of 100 kJ mol^–1^ nm^–2^. The REUS was then carried out for 2.5 μs using 64 windows spaced by 0.05 nm. This window spacing was selected to ensure sufficiently high exchange probabilities (e.g., >0.2). The convergence analysis is reported in Figure S23. In Figure 6, we plotted the free energy profiles obtained from the second halves of the umbrella trajectories. The force constants used for the coarse-grained REUS are listed in Equation S14 in the Supporting Information. WHAM^54^ was then used to reconstruct the free energy profiles for the second half of the trajectories.

### Free Energy Calculations of Fragment Interactions with Added Hydrogen-Bonding Terms in the Martini 3 Force Field

In addition, the Martini 3 force field, in combination with the OLIVES and/or Martini 3 with an extra hydrogen bond adopted from the OLIVES parameters for the quaternary bonds, was used to study the free energy profiles of the aforementioned fragments. To fine-tune the intermolecular interactions in the Martini 3 model, we used the OLIVES^32^ quaternary bond parameter for the Asn702–Asn702 side-chain interaction. The OLIVES LJ parameters for the Asn702–Asn702 side chain are as follows: σ = 0.39630469 nm and ϵ = 22.76096 kJ/mol. We added these LJ values on top of the Martini 3 parameters for the Asn702–Asn702 side-chain interaction. To do this, we defined a virtual site with these LJ parameters and placed it on the Asn702 side-chain center of mass. The simulation setup employed was identical to that used for the Martini 3 simulations. The REUS was then carried out for 900 ns using 64 windows spaced by 0.05 nm. The convergence analysis is reported in Figure S24. The force constants used for the coarse-grained REUS are listed in Equation S14 in the Supporting Information. WHAM^54^ was then used to reconstruct the free energy profiles for the second halves of the trajectories.

## Supplementary Material



## Notes

Contact probability for PFKL and PFKP monomers for each replica with random initial positions in Martini 3; contact probability per residue for PFKL replicas from random initial positions in Martini 3; contact probability per residue for PFKP replicas from random initial positions in Martini 3; possible native contact map scenarios for the formation of the PFKL filament; average contact maps and contact probability for PFKL and PFKP monomers in Martini 3; contact probability for PFKL and PFKP monomers in Martini 3 for each replica; average contact probability per residue for PFKL and PFKP in Martini 3; contact probability per residue for PFKL replicas in Martini 3; contact probability per residue for PFKP replicas in Martini 3; stability of PFKL filaments in different Martini force fields; short-range Coulomb and LJ interactions for PFKL and PFKP in different Martini force fields; contact probability for PFKL and PFKP monomers in OPEPv7 for each replica; contact probability per residue for PFKL replicas in OPEPv7; contact probability per residue for PFKP replicas in OPEPv7; atomistic representation of PFKP interface 1–Interface 1 contact; Interface 1–Interface 2 contacts in the PFKL R-state filament cryoEM structure; analysis of the PFKL fragment interface regions; free energy profiles of Interface 1–Interface 1 interaction for native PFKL and its N702T mutant using Martini 3 with the OLIVES model; analysis of the lowest minimum of the free energy in the Martini 3 force field with an extra hydrogen bond; average normalized number of contacts and contact probability for the PFKL monomer in scaled Martini 3 with an added hydrogen-bonding term; convergence analysis for the AMBER ff19SB force field; convergence analysis for the CHARMM36m force field; convergence analysis for the Martini 3 force field; convergence analysis for the Martini 3 force field with an added hydrogen-bonding term; residue comparison at Interface 1 and Loop 1 between PFKL and PFKP; residue comparison at Interface 1 and Loop 2 between PFKL and PFKP; differences in side-chain volume and in chemical character in PFKL and PFKP Interface 1 and Loop 1; differences in side-chain volume and in chemical character in PFKL and PFKP Interface 1 and Loop 2; statistical difference in side-chain volume and in chemical character in PFKL and PFKP Interface 1 and Loop 1; statistical difference in side-chain volume and in chemical character in PFKL and PFKP Interface 1 and Loop 2; and comparison of residue contact number fractions in all-atom and coarse-grained simulations of PFKL and PFKP Interface 1, Loop 1, and Loop 2 (PDF)

## †

Université Paris Cité, CNRS, ITODYS, F-75013, 15 rue Jean Antoine de Baïf, 75013 Paris, France
