# Supplementary material for: Multiscale Simulation of Phosphofructokinase‑1 Assemblies: Capturing the Interplay between Specific and Transient Interactions
Source: J Phys Chem B. 2025 Nov 17;129(47):12098–109. doi: 10.1021/acs.jpcb.5c05346 (PMC12670391; doi:10.1021/acs.jpcb.5c05346)
Supplement: Supplementary file 1 [file jp5c05346_si_001.pdf]

Supporting Information

Multiscale Simulation of Phosphofructokinase-1 Assemblies: Capturing the Interplay Between Specific and Transient Interactions

Mehrnoosh Khodam Hazrati[†], Tom Miclot[†,‡], and Stepan Timr[*,†,‡]

[†] J. Heyrovsky Institute of Physical Chemistry, Czech Academy of Sciences, Dolejskova 2155/3, 182 00 Prague 8, Czech Republic

[‡] Present address: Université Paris Cité, CNRS, ITODYS, F-75013, 15 rue Jean Antoine de Baïf, 75013 Paris, France

E-mail: stepan.timr@jh-inst.cas.cz

With a good approximation, we could assume that free tetramers and filaments formed by two tetramers are in equilibrium in the studied system, neglecting the higher orders of filaments:

$$2\mathrm{T} \rightleftharpoons 1\mathrm{F} \tag{S1}$$

Thus, the reaction quotient Q is as follows:

$$Q = \frac{[F]}{[T]^2} \tag{S2}$$

We calculated the free energy difference between the native (wild-type) and mutant PFKL from experimental data[8] based on the following relationship between ΔG and the reaction quotient Q:

$$\Delta G = \Delta G^\circ + RT \ln Q \tag{S3}$$

At equilibrium ΔG = 0 and the equation S3 becomes:

$$\Delta G^\circ = -RT \ln Q \tag{S4}$$

The monomer solution concentrations used in the mass photometry experiments[8] were 25 nM and 12.5 nM for native PFKL and the PFKL-N702T mutant, respectively. The native PFKL monomers predominantly formed filaments, with 60% of the protein in a filament and 20% in tetrameric assemblies, while the PFKL-N702T was 90% tetrameric and only $< 0.1\%$ in assemblies larger than tetramers. Using these data, we were able to estimate the free energy difference between native and mutant PFKL as follows:

$$[T_{native}]^2 = [\frac{0.2 \times 25 \times 10^{-9}}{4}]^2 = 1.563 \times 10^{-18} \tag{S5}$$

$$[T_{mutant}]^2 = [\frac{0.9 \times 12.5 \times 10^{-9}}{4}]^2 = 7.910 \times 10^{-18} \tag{S6}$$

$$[F_{native}] = \frac{0.6 \times 25 \times 10^{-9}}{8} = 1.875 \times 10^{-9} \tag{S7}$$

$$[F_{mutant}] = \frac{0.001 \times 12.5 \times 10^{-9}}{8} = 1.563 \times 10^{-12} \tag{S8}$$

$$Q_{native} = \frac{1.875 \times 10^{-9}}{1.5625 \times 10^{-18}} = 1.2 \times 10^{9} \tag{S9}$$

$$Q_{mutant} = \frac{1.563 \times 10^{-12}}{7.910 \times 10^{-18}} = 1.97 \times 10^{5} \tag{S10}$$

$$\Delta G^{\circ}_{native} = -2.479 \ln(1.2 \times 10^{9}) = -51.825 \quad \mathrm{kJ \cdot mol^{-1}} \tag{S11}$$

$$\Delta G^{\circ}_{mutant} = -2.479 \ln(1.97 \times 10^{5}) = -30.221 \quad \mathrm{kJ \cdot mol^{-1}} \tag{S12}$$

$$\Delta G^{\circ}_{native} - \Delta G^{\circ}_{mutant} = -21.604 \quad \mathrm{kJ \cdot mol^{-1}} \tag{S13}$$

$$f_c = \begin{cases} 3000 & 3.50 \le dis \le 4.50 \\ 2000 & 4.50 \le dis \le 5.00 \\ 1800 & 5.00 \le dis \le 5.50 \\ 1500 & 5.50 \le dis \le 6.00 \end{cases} \tag{S14}$$

Where $\mathrm{f_c}$ is the biased force constant ($\mathrm{kJ \cdot mol^{-1}}$) and dis is the distance between the center of mass (COM) of the two fragments (nm).

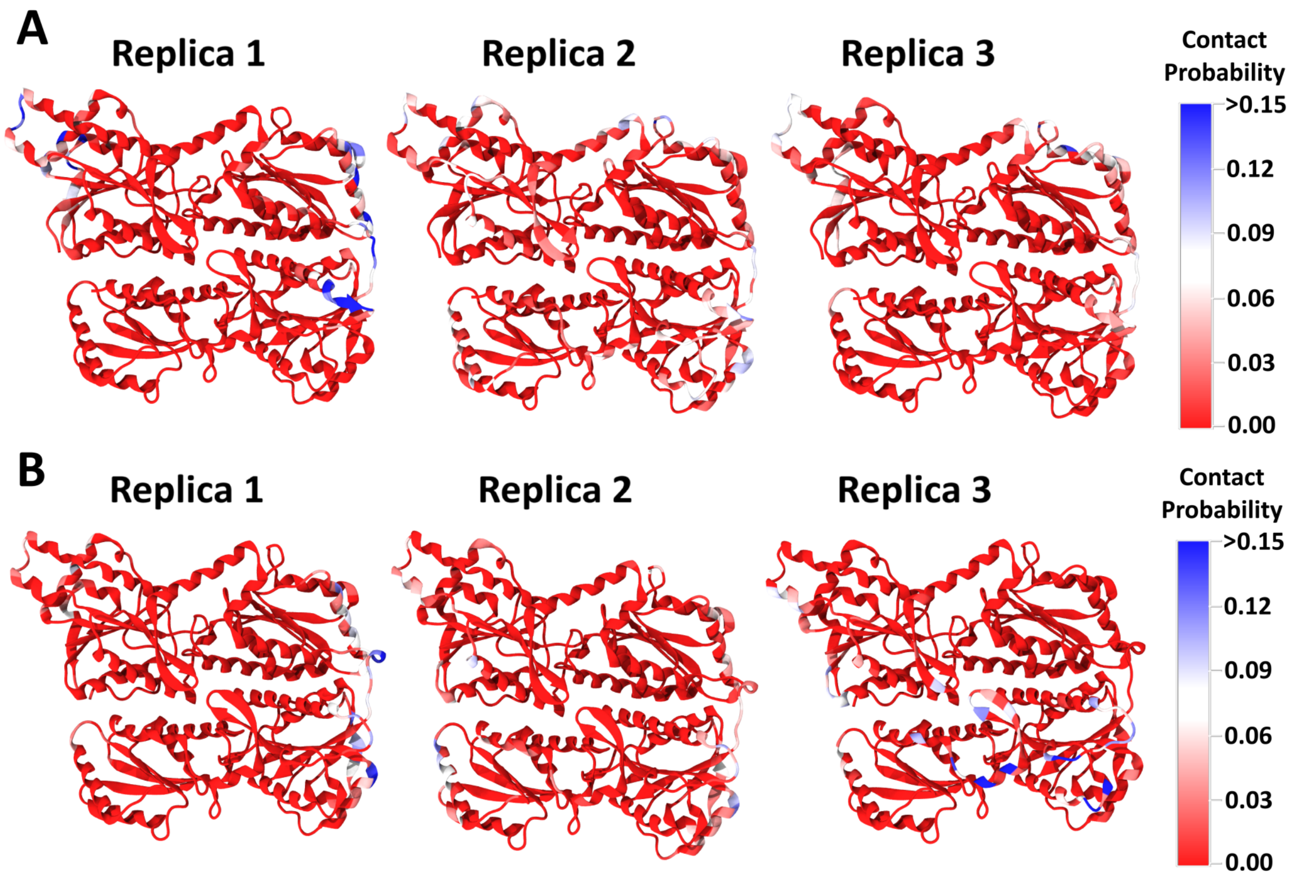

Figure S1: The contact probability for (A) PFKL and (B) PFKP monomer for each replica with random initial positions in Martini 3. Beads are considered to be in contact if the distance between them is less than 0.7 nm. The values from coarse-grained simulations are averaged over the eight monomers and mapped to the CryoEM structures. The color bar shows the contact probability during the simulation time (15 μs): blue means the residue is in contact with the other tetramer, while red means that there is no contact.

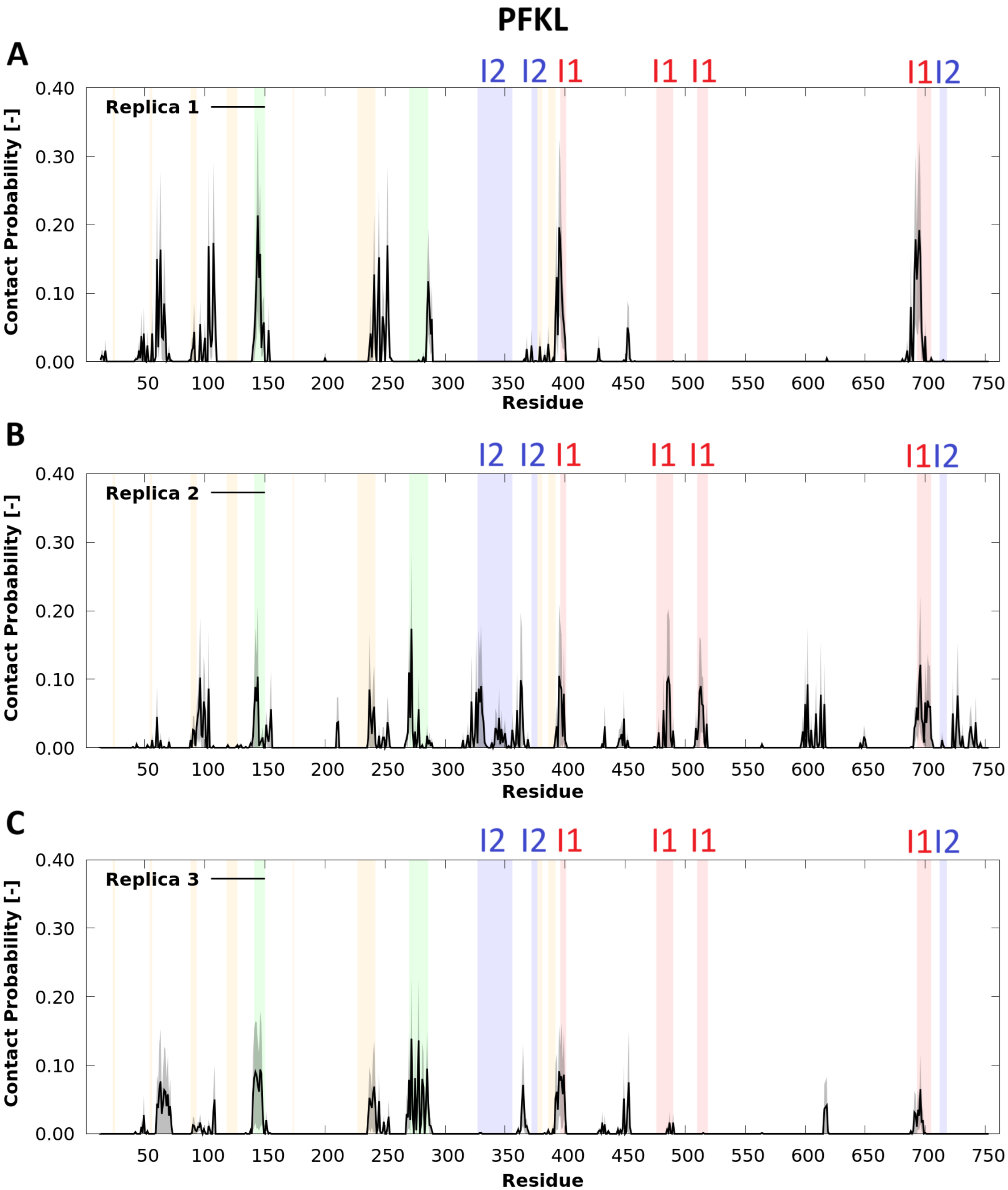

Figure S2: The contact probability per residue for PFKL replicas from random initial positions in Martini 3. The shaded areas in gray show the standard error of the mean (SEM). The values are averaged over the eight monomers. The residue ranges of Interface 1 and Interface 2 are highlighted in red and blue, respectively. The shaded orange and green regions are the ATP binding sites and two important epitope regions, respectively.

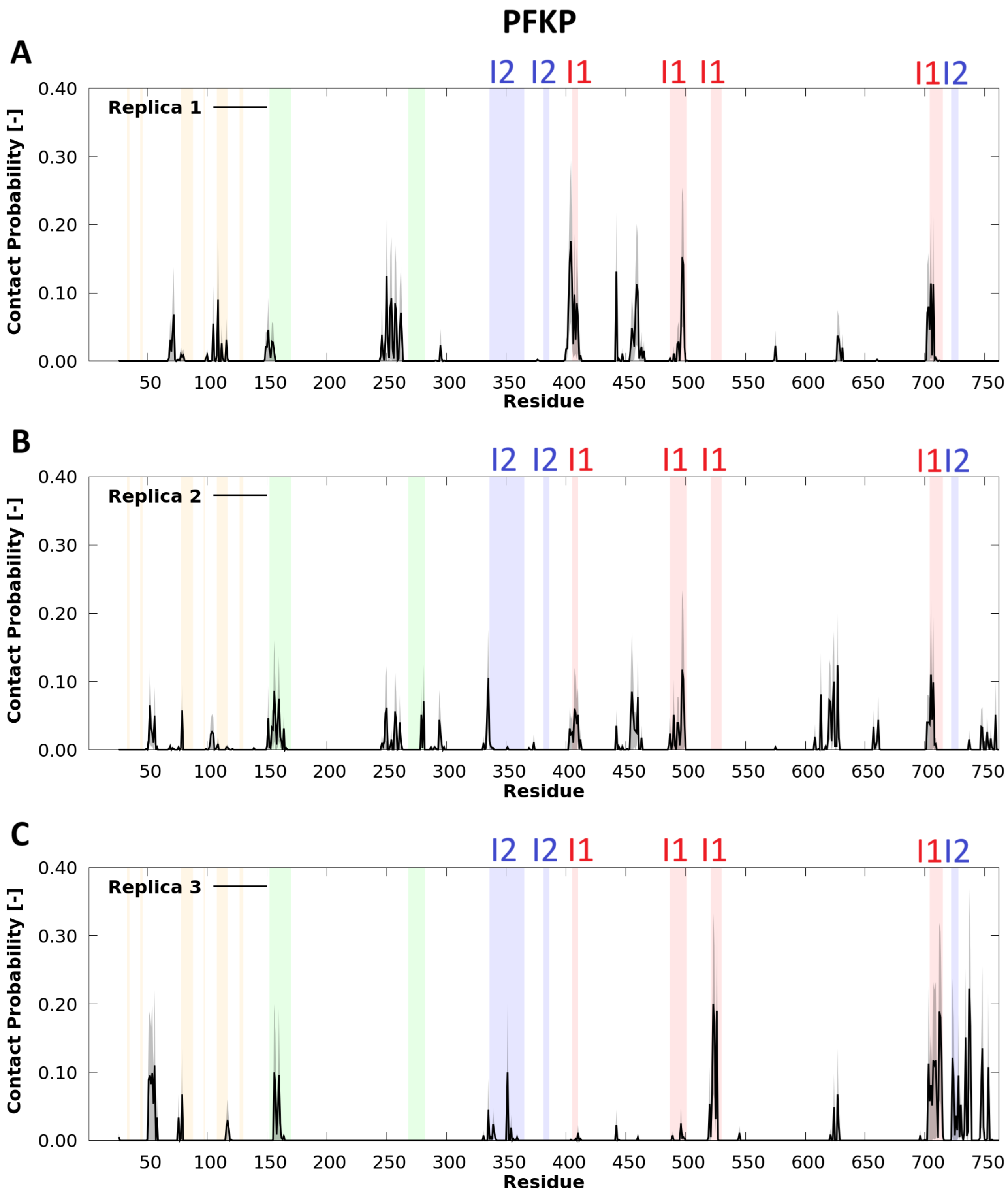

Figure S3: The contact probability per residue for PFKP replicas from random initial positions in Martini 3. The shaded areas in gray show the standard error of the mean (SEM). The values are averaged over the eight monomers. The residue ranges of Interface 1 and Interface 2 are highlighted in red and blue, respectively. The shaded orange and green regions are the ATP binding sites and two important epitope regions, respectively.

Figure S4 illustrates the possible native contact scenarios for PFKL filament formation. Figure S5B and C show the average contact maps for the PFKL and PFKP tetramer–tetramer interactions within the Martini 3 model starting from initial preformed filament configurations. In the case of PFKL, the two interactions, Interface 1–Interface 2, are maintained for the duration of 15 μs of coarse-grained simulation time, while Interface 1–Interface 1 remains in contact for approximately 50%. The formation of the PFKL filament is consistent with the second possibility illustrated in Figure S4, which is a consequence of the initial configuration. Martini 3 predicted a stable filament for PFKP by maintaining all three interaction sites during the 15 μs of coarse-grained simulation time. Figure S5D and E show the average contact probability for PFKL and PFKP tetramer backbone beads over 3 replicas starting from the preformed filament structure in Martini 3. Figure S6A and B show the contact probability for PFKL and PFKP tetramer backbone beads for each replica. The average contact probabilities per residue for PFKL and PFKP are shown in Figure S7. Mostly, the interactions occur at the interfaces, which is a consequence of the initial preformed filament configuration. The contact probabilities per residue for each replica of PFKL and PFKP are shown in Figure S8 and Figure S9, respectively.

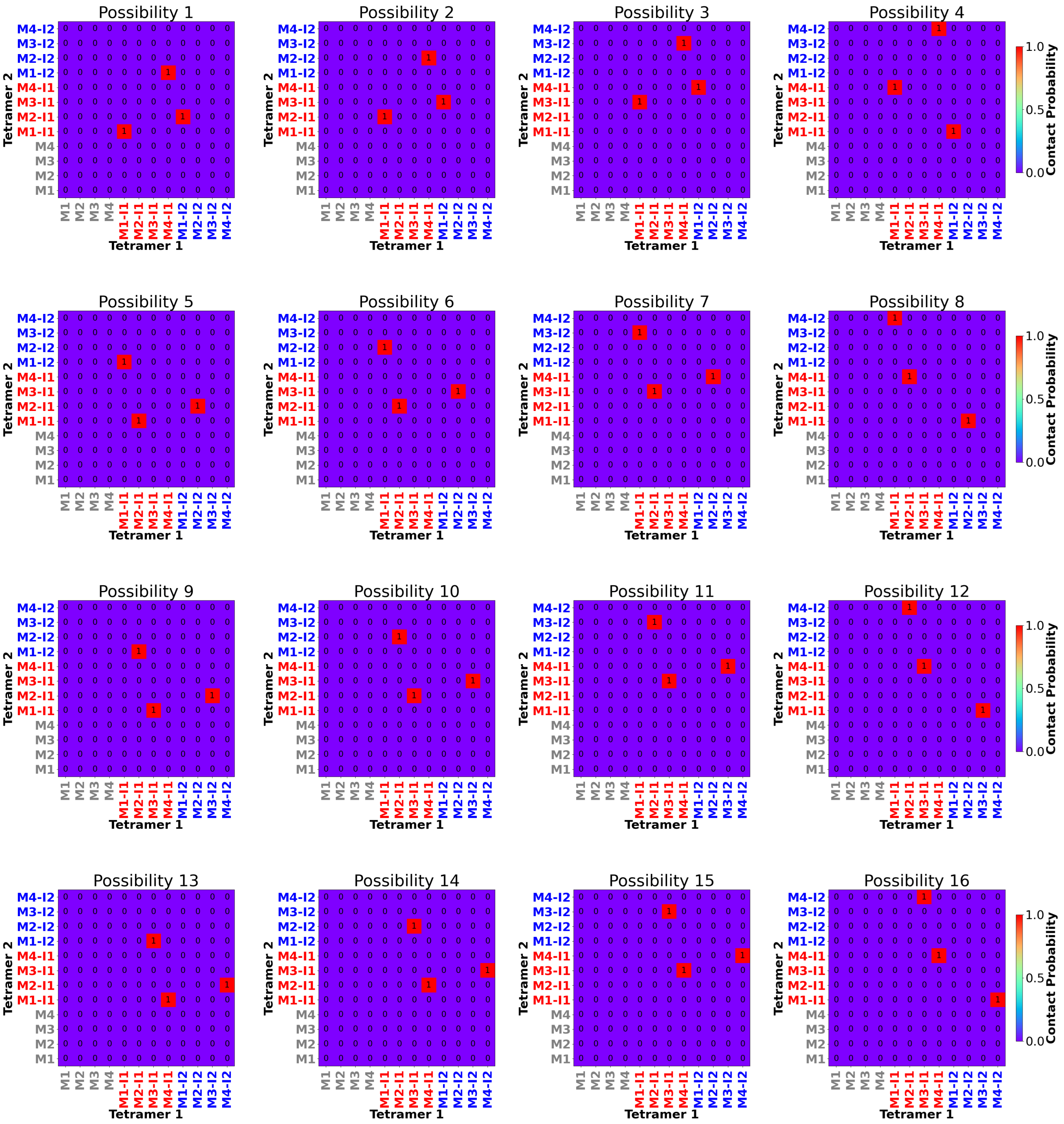

Figure S4: The maps illustrate the possible native contact scenarios for PFKL filament formation. For a PFKL filament to form, three interaction sites must be in contact. For two tetramers, a total of 16 possibilities are available.

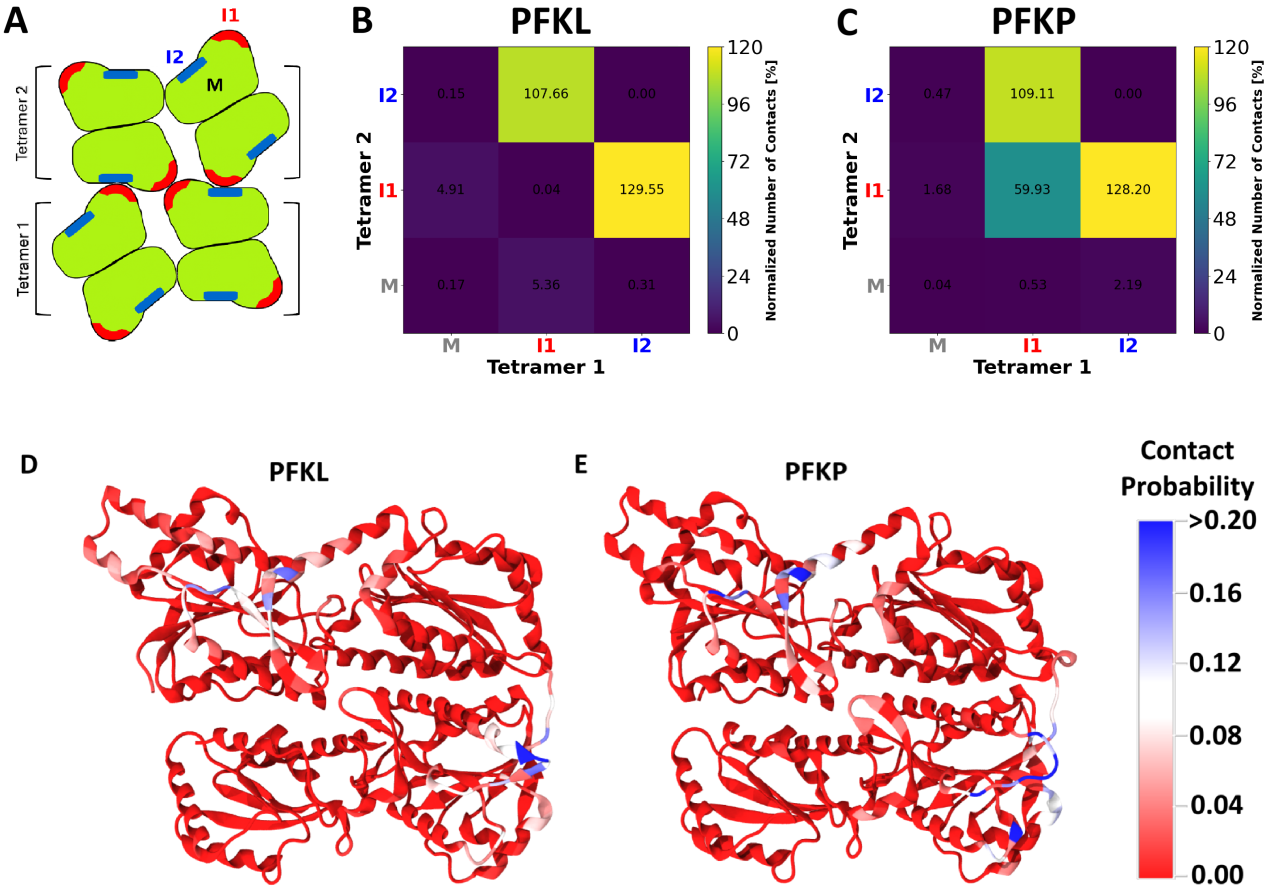

Figure S5: Interactions between PFK1 tetramers in Martini 3 simulations starting from initial preformed filament configurations. (A) A representative cartoon of the filament structure for PFKL and PFKP. Individual monomers forming each tetramer are shown as a green surface. The red regions show Interface 1, while the blue regions indicate Interface 2 in each monomer. Average contact maps of (B) PFKL and (C) PFKP tetramer–tetramer interactions. The numbering of monomers and interfaces in (A) is used. M1 to M4 indicate the monomers in each tetramer, excluding the residues of Interfaces 1 and 2. The average contact probability for (D) PFKL and (E) PFKP monomers over 3 replicas. Beads are considered to be in contact if the distance between them is less than 0.7 nm. The values from coarse-grained simulations are averaged over the eight monomers and mapped to the CryoEM structures. The color bar shows the contact probability during the simulation time (15 μs): blue means the residue is in contact with the other tetramer, while the red indicates that no contact with the other tetramer has occurred, or in other words, the distance between the bead and the other tetramer has remained greater than 0.7 nm throughout the simulation.

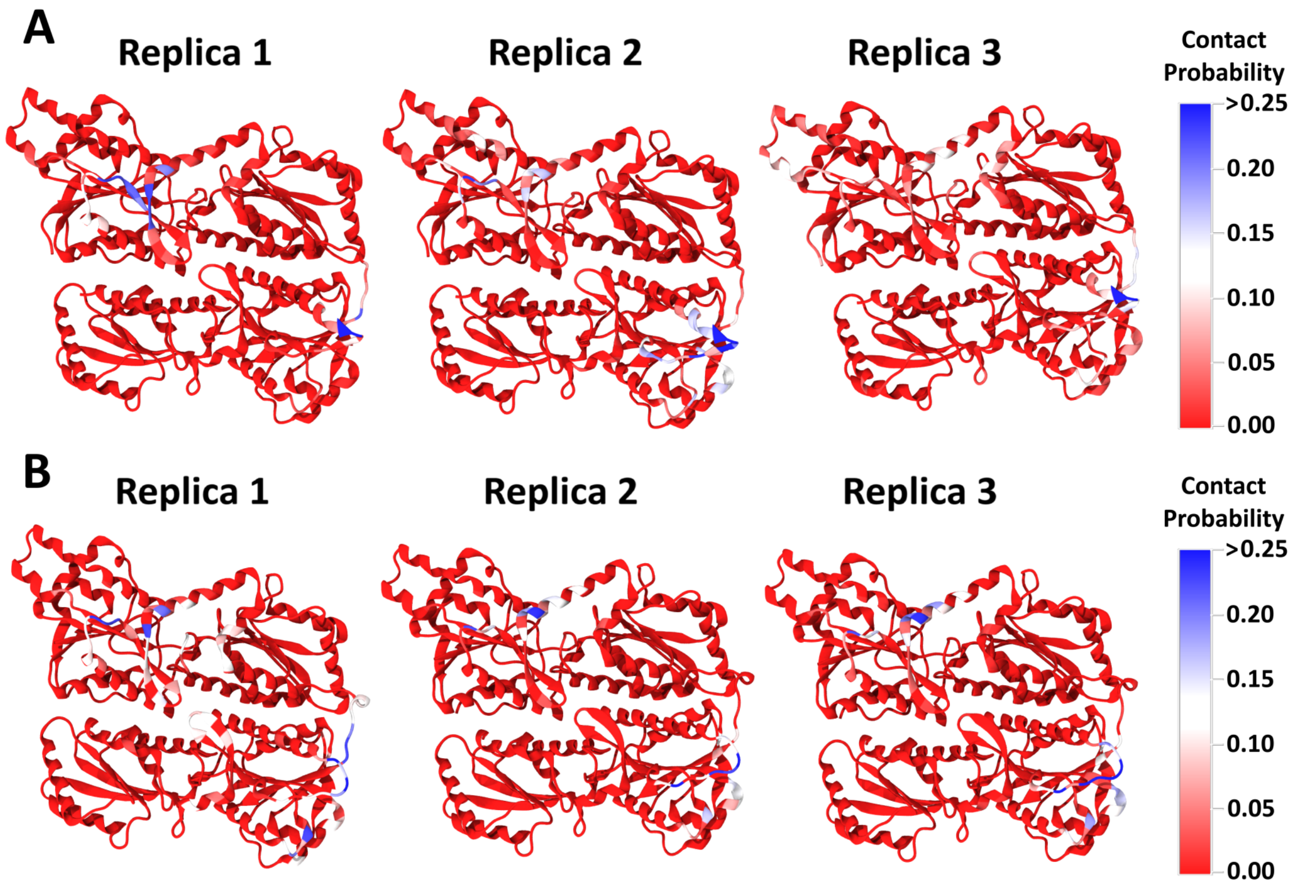

Figure S6: The contact probability for (A) PFKL and (B) PFKP monomer for each replica with initial preformed filament structures in Martini 3. Beads are considered to be in contact if the distance between them is less than 0.7 nm. The values from coarse-grained simulations are averaged over the eight monomers and mapped to the CryoEM structures. The color bar shows the contact probability during the simulation time (15 μs): blue means the residue is in contact with the other tetramer, while red means that there is no contact.

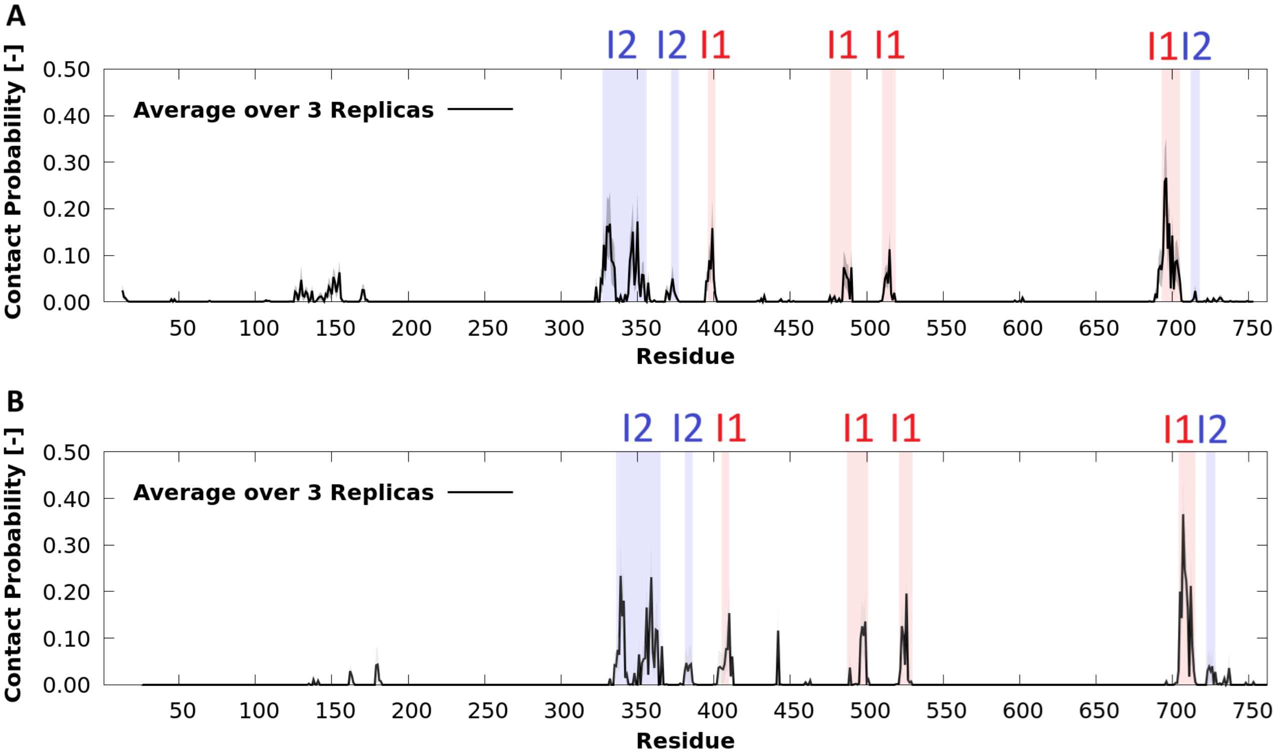

Figure S7: The average contact probability per residue for (A) PFKL and (B) PFKP in Martini 3 from initial preformed filament configurations over eight monomers and three replicas. The shaded areas in gray show the standard error of the mean (SEM). The residue ranges of Interface 1 and Interface 2 are highlighted in red and blue, respectively.

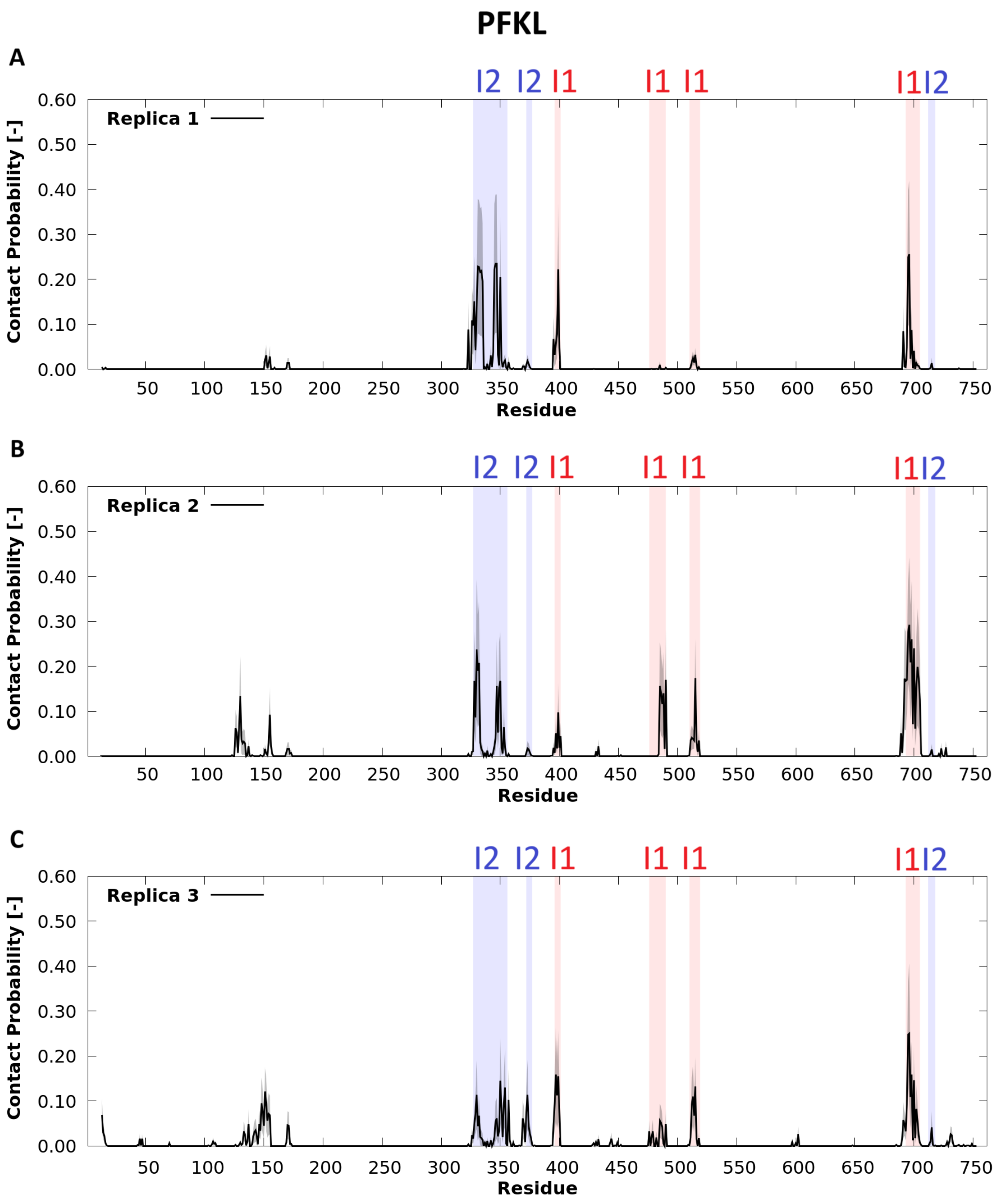

Figure S8: The contact probability per residue for PFKL replicas from initial preformed filament configurations in Martini 3. The shaded areas in gray show the standard error of the mean (SEM). The values are averaged over the eight monomers. The residue ranges of Interface 1 and Interface 2 are highlighted in red and blue, respectively.

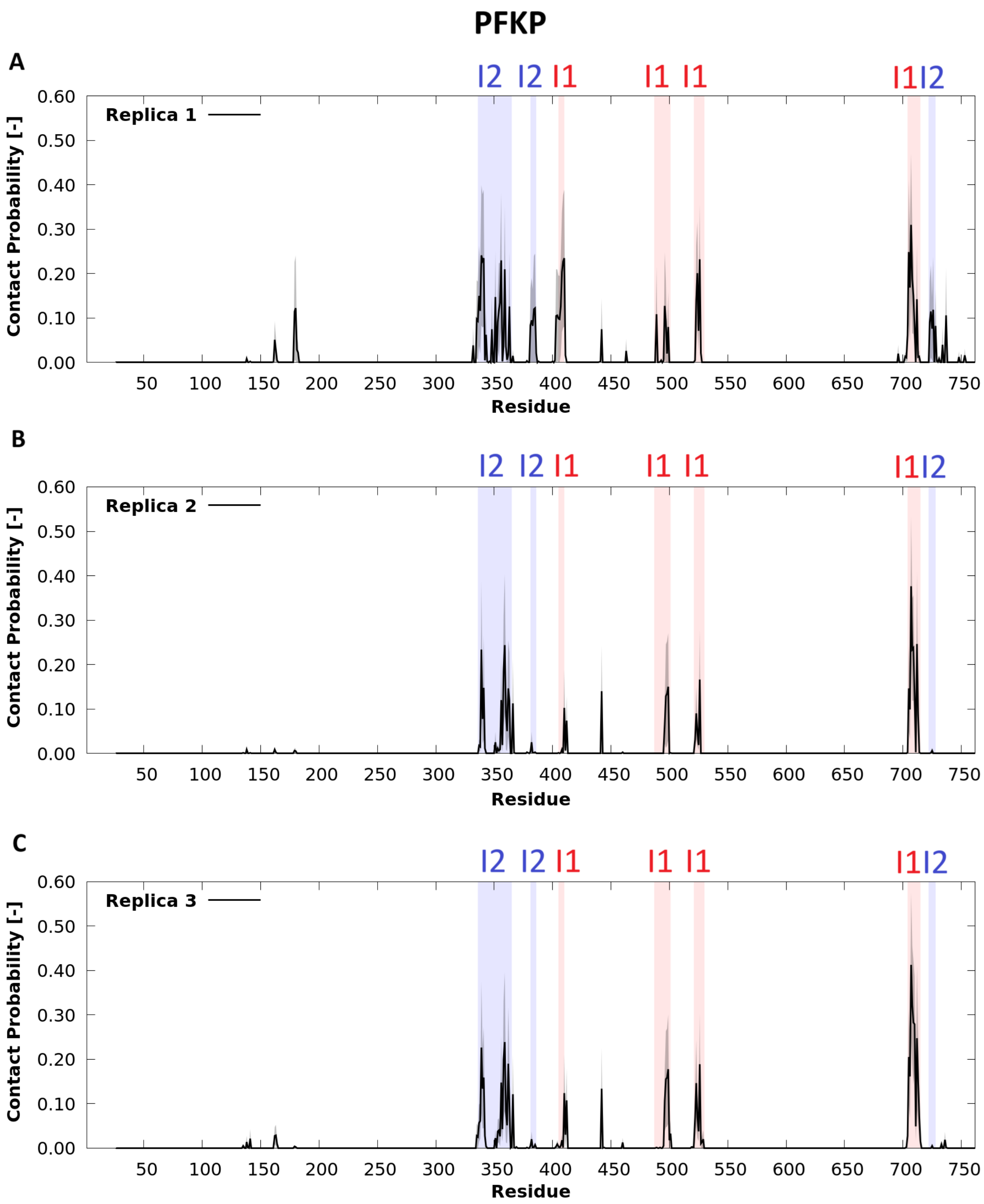

Figure S9: The contact probability per residue for PFKP replicas from initial preformed filament configurations in Martini 3. The shaded areas in gray show the standard error of the mean (SEM). The values are averaged over the eight monomers. The residue ranges of Interface 1 and Interface 2 are highlighted in red and blue, respectively.

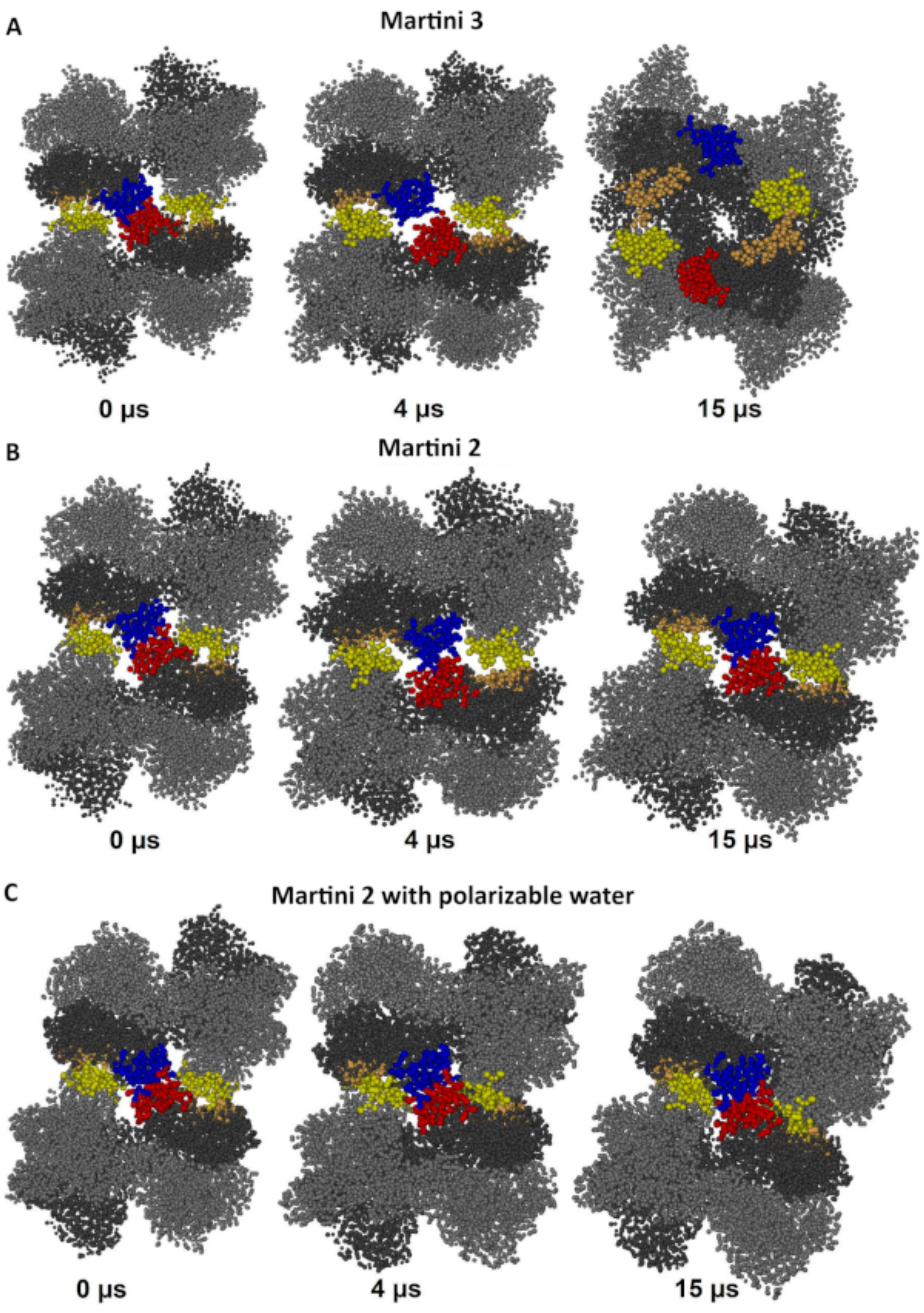

Figure S10: The stability of PFKL filaments during 15 μs of coarse-grained simulations with (A) Martini 3, (B) Martini 2, and (C) Martini 2 with polarizable water model. For enhanced visual representation, the two Interfaces 1 in contact are depicted in red and blue. Interface 1 and Interface 2 in contact are illustrated in yellow and orange, respectively. The remaining beads of monomers are colored in dark and light gray.

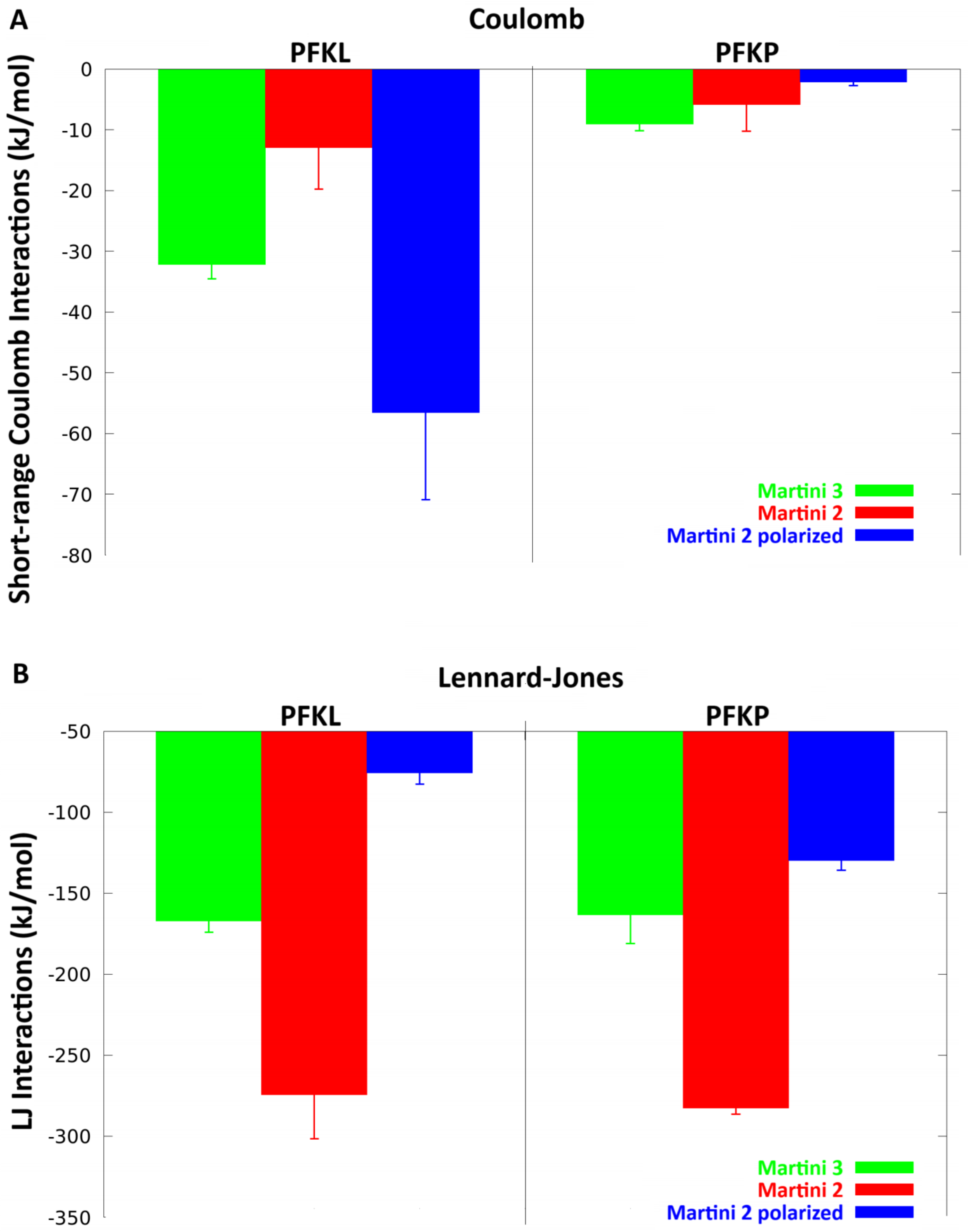

Figure S11: (A) Short-range Coulomb and (B) LJ interactions between Interface 1 of two tetramers for PFKL and PFKP using different Martini force fields. The values are averaged over 3 replicas after a short equilibration. The error bars represent the standard deviations.

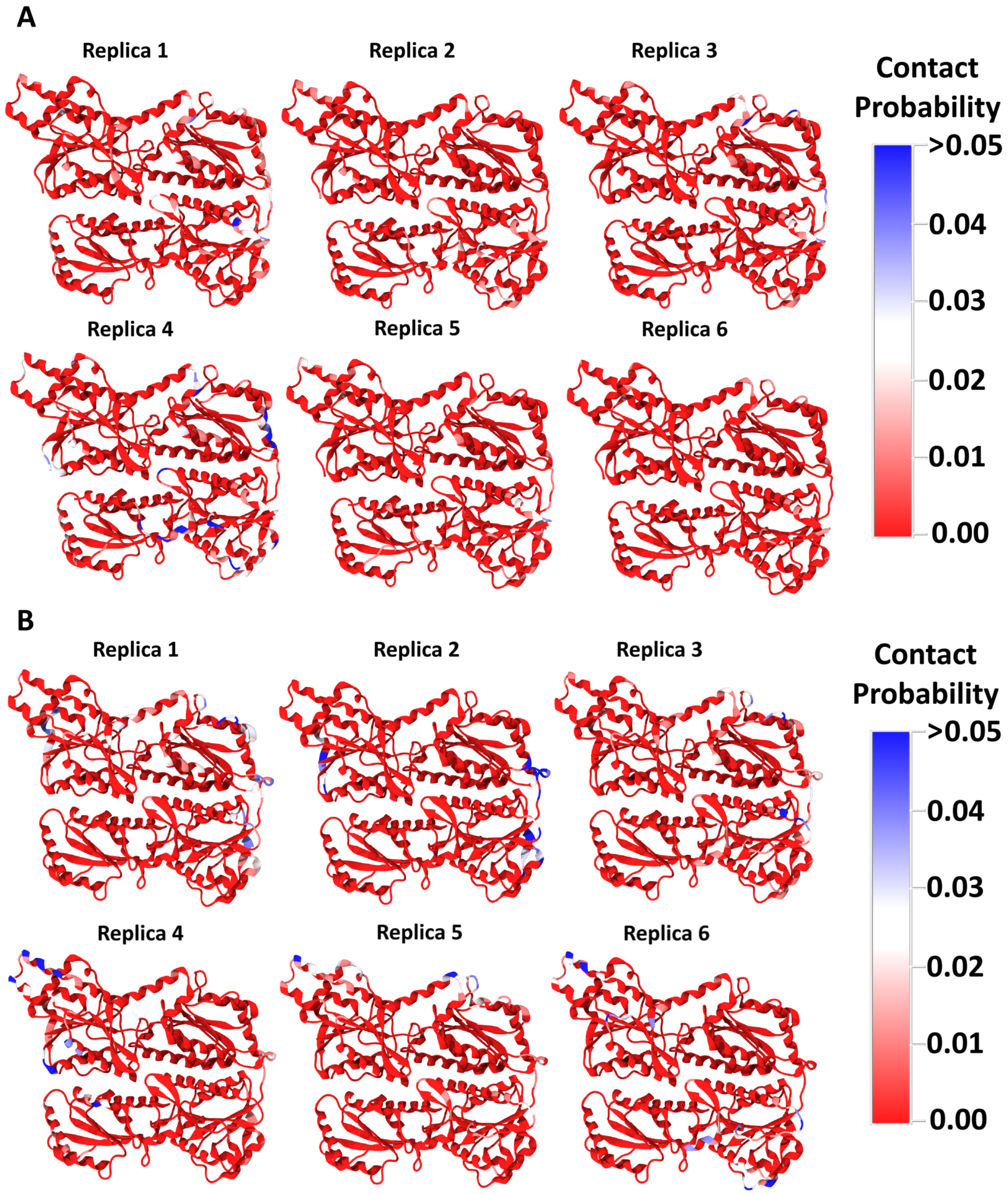

Figure S12: The contact probability for (A) PFKL and (B) PFKP tetramer beads for each replica in OPEPv7. Beads are considered to be in contact if the distance between them is less than 0.7 nm. The values from coarse-grained simulations are averaged over the eight monomers and mapped to the CryoEM structures. The color bar shows the contact probability during the simulation time (150 μs): blue means the bead is in contact with the other tetramer, while red means that there is no contact.

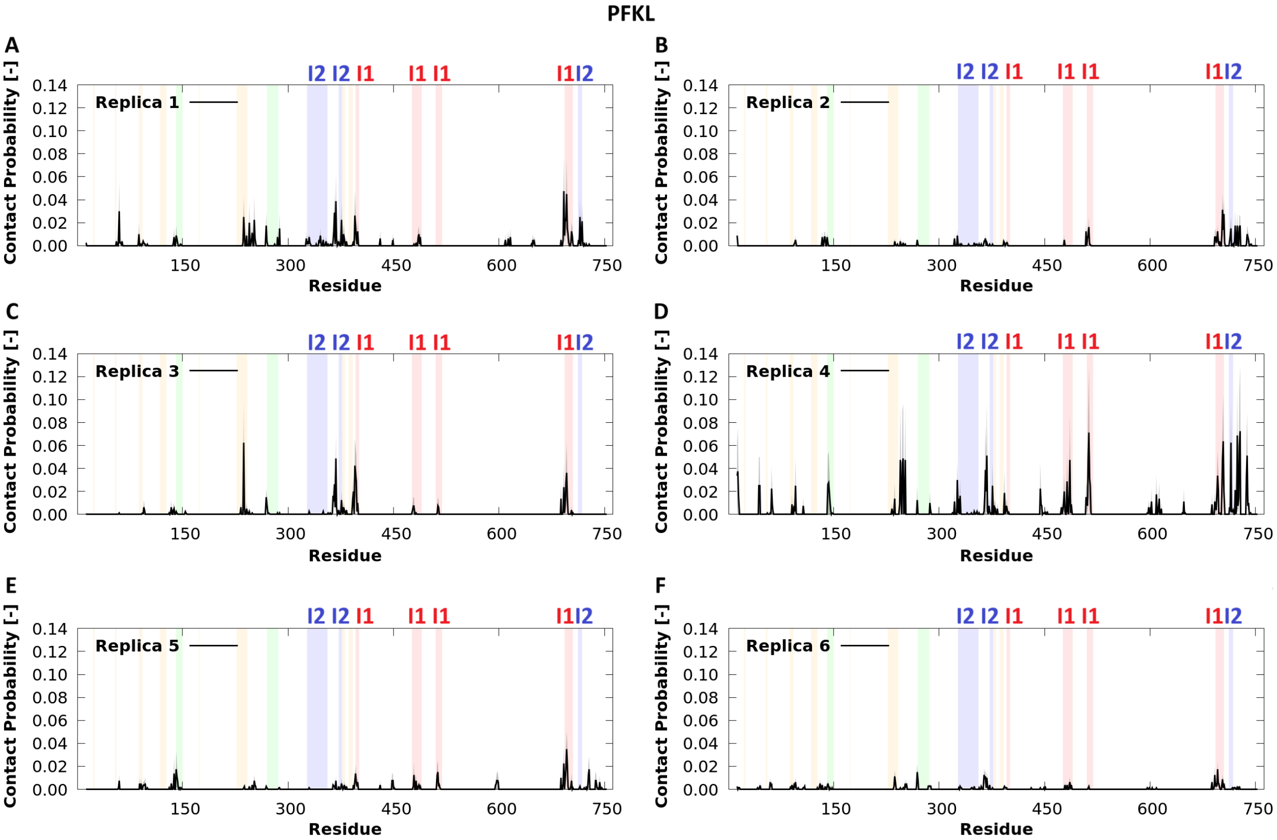

Figure S13: The contact probability per residue for PFKL replicas in OPEPv7. The shaded areas in gray show the standard error of the mean (SEM). The values are averaged over the eight monomers. The residue ranges of Interface 1 and Interface 2 are highlighted in red and blue, respectively. The shaded orange and green regions are the ATP binding sites and two important epitope regions, respectively.

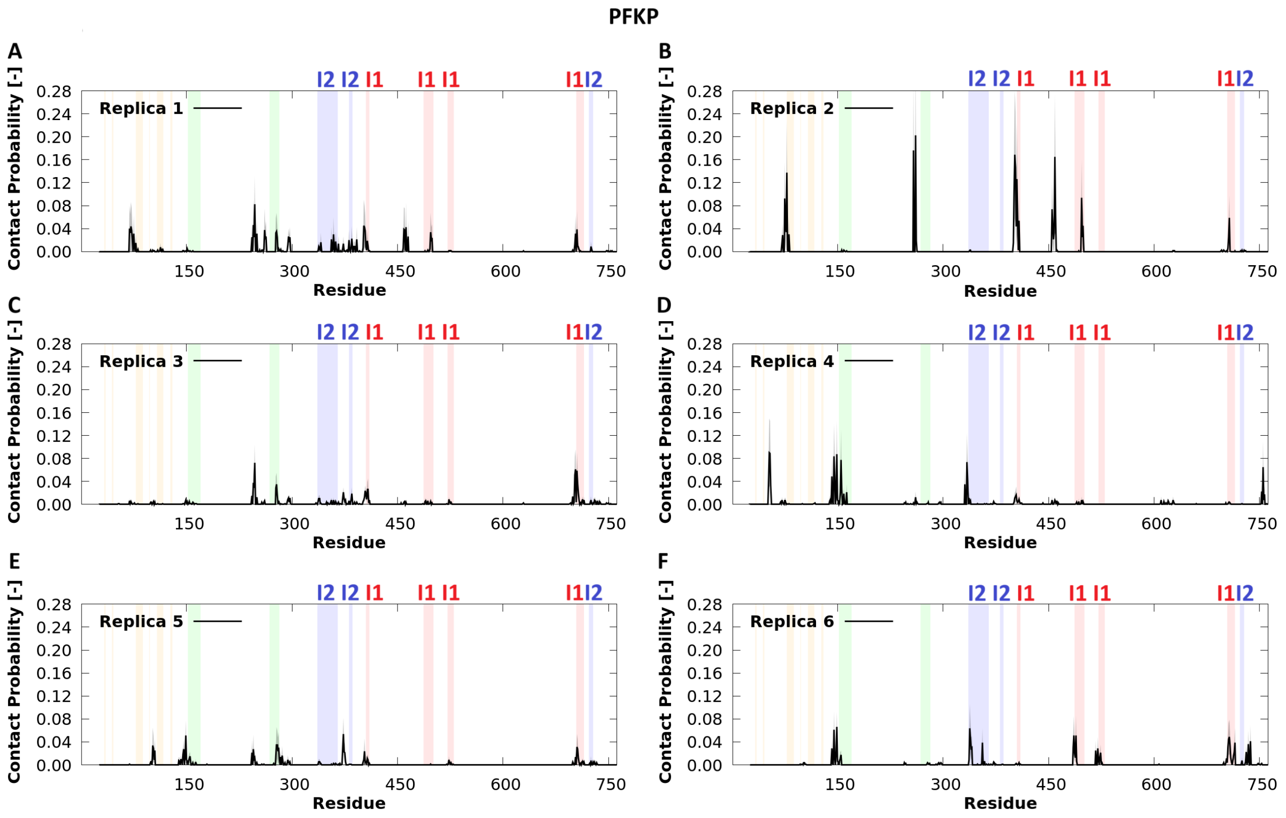

Figure S14: The contact probability per residue for PFKP replicas in OPEPv7. The shaded areas in gray show the standard error of the mean (SEM). The values are averaged over the eight monomers. The residue ranges of Interface 1 and Interface 2 are highlighted in red and blue, respectively. The shaded orange and green regions are the ATP binding sites and two important epitope regions, respectively.

Comparative Sequence and Residue Analysis of Interface 1, Loop 1 and Loop 2, in PFKL and PFKP

A residue-by-residue comparison of PFKL and PFKP Interface 1, Loop 1 and 2 is shown in Table S1 and Table S2, respectively. The comparison of Loop 1 and Loop 2 sequences in PFKL and PFKP revealed that Loop 2 has a substantially higher number of substitutions, with differences both in side-chain volume and in chemical character (see Table S3 - Table S6). Statistical analysis of residue properties shows that in Loop 2, PFKP has a higher proportion of charged residues as well as polar and hydrophilic side chains compared to PFKL. Loop 1 also follows this trend, though to a lesser extent. The enrichment of charged residues in PFKP could facilitate the formation of multiple transient interactions at Interface 1, consistent with the occasional, short-lived contacts observed in our simulations.

Nevertheless, the ability to establish transient, charge-mediated contacts does not directly translate into filament formation. Filament stability requires not only intermolecular attraction but also a precise complementarity of residues and a defined internal organization of the interface.

Table S1: Residue comparison at Interface 1, Loop 1 between PFKL and PFKP. Code = conserved residues, * = significant difference, + = conserved feature.

Interface 1, Loop 1										
**PFKL**	510	511	512	513	514	515	516	517	518	519
	Ala	Arg	Gly	Arg	Tyr	Glu	Glu	Leu	Cys	Ile
	A	R	*	+	+	E	E	*	C	+
**PFKP**	521	522	523	524	525	526	527	528	529	530
	Ala	Arg	Glu	Lys	His	Glu	Glu	Phe	Cys	Val

Table S2: Residue comparison at Interface 1, Loop 2 between PFKL and PFKP. Code = conserved residues, * = significant difference, + = conserved feature.

Interface 1, Loop 2													
**PFKL**	693	694	695	696	697	698	699	700	701	702	703	704	705
	Val	Tyr	Arg	Lys	Gly	Arg	Val	Phe	Ala	Asn	Ala	Pro	Asp
	*	*	*	+	G	+	*	F	—	*	*	*	D
**PFKP**	704	705	706	707	708	709	710	711	—	712	713	714	715
	Ala	Arg	Gly	Arg	Gly	Lys	Lys	Phe	—	Thr	Thr	Asp	Asp

Table S3: Differences in side-chain volume and in chemical character in PFKL and PFKP Interface 1, Loop 1.

**Position**	**PFKL Residue**	**PFKP Residue**	**Volume**	**Polarity**	**Hydropathy**	**Charge**	**Chemical Class**
1	Ala	Ala	Same	Same	Same	Same	Same
2	Arg	Arg	Same	Same	Same	Same	Same
3	Gly	Glu	VS→M	NP→P	N→Hphi	UnCh→Ch	BB→A
4	Arg	Lys	Same	Same	Same	Same	Same
5	Tyr	His	VL→M	Same	Same	UnCh→Ch	Aro→B
6	Glu	Glu	Same	Same	Same	Same	Same
7	Glu	Glu	Same	Same	Same	Same	Same
8	Leu	Phe	L→VL	Same	Same	Same	Ali→Aro
9	Cys	Cys	Same	Same	Same	Same	Same
10	Ile	Val	L→M	Same	Same	Same	Same

The classification is based on the paper by Pommie et al.[25] Abbreviations used in the table: VS = Very Small; S = Small, M = Medium, L = Large; VL = Very Large; P = Polar; NP = Nonpolar; BB = Backbone; A = Acidic; B = Basic; Aro = Aromatic; N = Neutral; Ch = Charged; UnCh = Uncharged; Hphi = Hydrophilic; Hpho = Hydrophobic; H = Hydroxyl; Ali = Aliphatic; Same = no change; Gap = residue absent.

Table S4: Differences in side-chain volume and in chemical character in PFKL and PFKP Interface 1, Loop 2.

**Position**	**PFKL Residue**	**PFKP Residue**	**Volume**	**Polarity**	**Hydropathy**	**Charge**	**Chemical Class**
1	Val	Ala	M→VS	Same	Same	Same	Same
2	Tyr	Arg	VL→L	Same	N→Hphi	UnCh→Ch	Aro→B
3	Arg	Gly	L→VS	P→NP	Hphi→N	Ch→UnCh	B→BB
4	Lys	Arg	Same	Same	Same	Same	Same
5	Gly	Gly	Same	Same	Same	Same	Same
6	Arg	Lys	Same	Same	Same	Same	Same
7	Val	Lys	M→L	NP→P	Hpho→Hphi	UnCh→Ch	Ali→B
8	Phe	Phe	Same	Same	Same	Same	Same
9	Ala	—	VS→Gap	NP→Gap	Hpho→Gap	UnCh→Gap	Ali→Gap
10	Asn	Thr	Same	Same	Hphi→N	Same	Amide→H
11	Ala	Thr	VS→S	NP→P	Hpho→N	Same	Ali→H
12	Pro	Asp	Same	NP→P	N→Hphi	UnCh→Ch	BB→A
13	Asp	Asp	Same	Same	Same	Same	Same

The classification is based on the paper by Pommie et al.[25] Abbreviations used in the table: VS = Very Small; S = Small, M = Medium, L = Large; VL = Very Large; P = Polar; NP = Nonpolar; BB = Backbone; A = Acidic; B = Basic; Aro = Aromatic; N = Neutral; Ch = Charged; UnCh = Uncharged; Hphi = Hydrophilic; Hpho = Hydrophobic; H = Hydroxyl; Ali = Aliphatic; Same = no change; Gap = residue absent.

Table S5: Statistics difference in side-chain volume and in chemical character in PFKL and PFKP Interface 1, Loop 1.

**Volume**	**VS**	**S**	**M**	**L**	**VL**				**SUM**
**Number of residues**	-1	0	3	-2	0				0
**Polarity**	**P**	**NP**							**SUM**
**Number of residues**	1	-1							0
**Hydropathy**	**Hpho**	**N**	**Hphi**						**SUM**
**Number of residues**	0	-1	1						0
**Charge**	**Ch**	**UnCh**							**SUM**
**Number of residues**	2	-2							0
**Chemical class**	**Amide**	**A**	**B**	**H**	**S**	**Ali**	**Aro**	**BB**	**SUM**
**Number of residues**	0	1	1	0	0	-1	0	-1	0
**Fraction of mutated**									0.50
**Fraction of significantly different**									0.40

The classification is based on the paper by Pommie et al.[25] Abbreviations used in the table: VS = Very Small; S = Small, M = Medium, L = Large; VL = Very Large; P = Polar; NP = Nonpolar; BB = Backbone; A = Acidic; B = Basic; Aro = Aromatic; N = Neutral; Ch = Charged; UnCh = Uncharged; Hphi = Hydrophilic; Hpho = Hydrophobic; H = Hydroxyl; Ali = Aliphatic; S = Sulfur.

Table S6: Statistics difference in side-chain volume and in chemical character in PFKL and PFKP Interface 1, Loop 2.

**Volume**	**VS**	**S**	**M**	**L**	**VL**				**SUM**
**Number of residues**	0	1	-2	1	-1				-1
**Polarity**	**P**	**NP**							**SUM**
**Number of residues**	2	-3							-1
**Hydropathy**	**Hpho**	**N**	**Hphi**						**SUM**
**Number of residues**	-3	1	1						-1
**Charge**	**Ch**	**UnCh**							**SUM**
**Number of residues**	2	-3							-1
**Chemical class**	**Amide**	**A**	**B**	**H**	**S**	**Ali**	**Aro**	**BB**	**SUM**
**Number of residues**	-1	1	1	2	0	-3	-1	0	-1
**Fraction of mutated**									0.77
**Fraction of significantly different**									0.62

The classification is based on the paper by Pommie et al.[25] Abbreviations used in the table: VS = Very Small; S = Small, M = Medium, L = Large; VL = Very Large; P = Polar; NP = Nonpolar; BB = Backbone; A = Acidic; B = Basic; Aro = Aromatic; N = Neutral; Ch = Charged; UnCh = Uncharged; Hphi = Hydrophilic; Hpho = Hydrophobic; H = Hydroxyl; Ali = Aliphatic; S = Sulfur.

Table S7 shows the PFKL Interface 1-Interface 1 (Loop 1 and Loop 2) residues from the all-atom and coarse-grained trajectories (after 1 and 15 μs, averaged over three replicas). At Interface 1, Loop 1 and 2, PFKP contains 14 mutations (colored in red) compared to PFKL. Residues identified as possible targets for modification are highlighted in yellow. These residues differ from those in PFKP and also indicate the pairs most underestimated in coarse-grained compared to all-atom (differences greater than 0.3, where a value of 1 indicates that the residue pair was in contact throughout the entire simulation. We normalized the values for the coarse-grained simulations based on the maximum value.) Therefore, a total of 9 residues could be considered for modification. However, we selected Asn702 for minimal bead modification because it belongs to the Loop 2—the important region of the Interface 1—and it is experimentally recognized as a key residue in forming PFKL filaments, since the N702T mutation has been shown to disrupt filament formation. In principle, one could also consider the other 8 residues for additional fine-tuning.

Table S7: A comparison of residue contact number fractions in all-atom and coarse-grained simulations of PFKL and PFKP Interface 1, Loop 1 and Loop 2. The value of 1 indicates that the residue was in contact throughout the entire simulation with the other fragment. Abbreviations used in the table: AA = All-atom; CG = Coarse-grained; Diff = Difference

	**PFKL Residue Number**	**PFKL Residue Name**	**AA**	**CG (1 μs)**	**Diff**	**CG (15 μs)**	**Diff**	**PFKP Residue Name**	**PFKP Residue Number**
**Loop 1**	510	Ala	1.000	0.026	0.974	0.006	0.994	Ala	521
	511	Arg	1.000	0.285	0.715	0.074	0.926	Arg	522
	512	Gly	1.000	0.570	0.430	0.188	0.812	Glu	523
	513	Arg	1.000	0.673	0.327	0.218	0.782	Lys	524
	514	Tyr	1.000	0.557	0.443	0.147	0.853	His	525
	515	Glu	1.000	0.846	0.154	0.424	0.576	Glu	526
	516	Glu	1.000	0.240	0.760	0.066	0.934	Glu	527
	517	Leu	1.000	0.091	0.909	0.013	0.987	Phe	528
	518	Cys	1.000	0.310	0.690	0.072	0.928	Cys	529
	519	Ile	0.003	0.000	0.003	0.000	0.003	Val	530
**Loop 2**	693	Val	0.987	0.221	0.766	0.257	0.730	Ala	704
	694	Tyr	0.420	0.272	0.148	0.388	0.032	Arg	705
	695	Arg	1.000	1.000	0.000	0.967	0.033	Gly	706
	696	Lys	0.983	0.741	0.243	1.000	-0.017	Arg	707
	697	Gly	0.193	0.335	-0.142	0.427	-0.234	Gly	708
	698	Arg	1.000	0.749	0.251	0.634	0.366	Lys	709
	699	Val	0.887	0.180	0.706	0.108	0.779	Lys	710
	700	Phe	1.000	0.816	0.184	0.535	0.465	Phe	711
	701	Ala	0.997	0.273	0.724	0.100	0.897	—	—
	702	Asn	1.000	0.519	0.481	0.321	0.679	Thr	712
	703	Ala	1.000	0.434	0.566	0.330	0.670	Thr	713
	704	Pro	0.993	0.216	0.777	0.255	0.738	Asp	714
	705	Asp	0.687	0.182	0.505	0.169	0.518	Asp	715

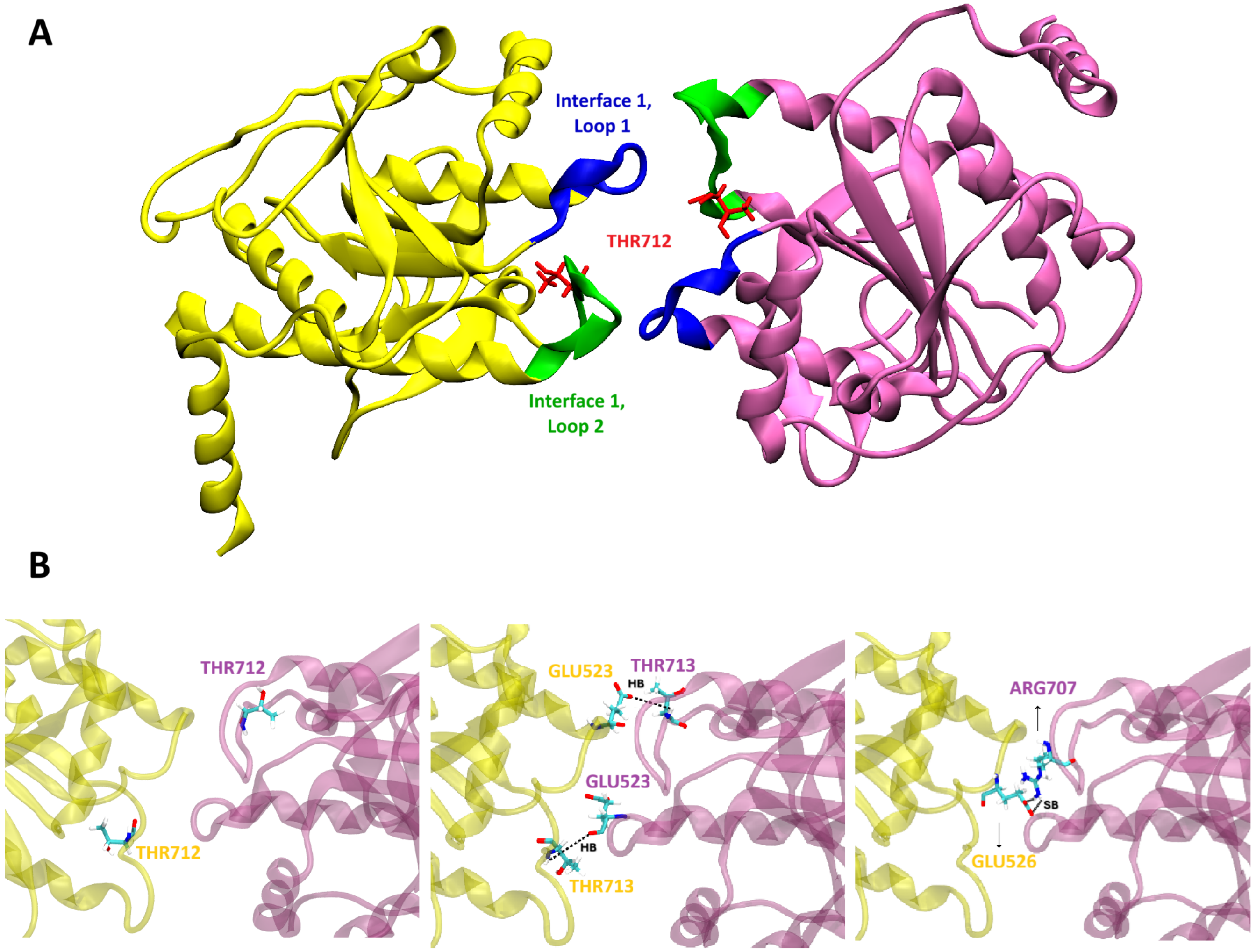

Figure S15: (A) Atomistic representation of the initial structures of the two PFKP fragments, aligned to PFKL Interface 1 structure obtained by CryoEM.[8] Fragments A and B are shown in yellow and magenta, and Loop 1 and Loop 2 of Interface 1 are shown in blue and green, respectively. The Thr712 residues of each fragment are highlighted in red. (B) Zoom-in view of Thr712 and the most interacting residues during three 500 ns replicas of atomistic MD simulations performed to relax the CryoEM structure. SB stands for ”salt bridge”, and HB stands for ”hydrogen bond.”

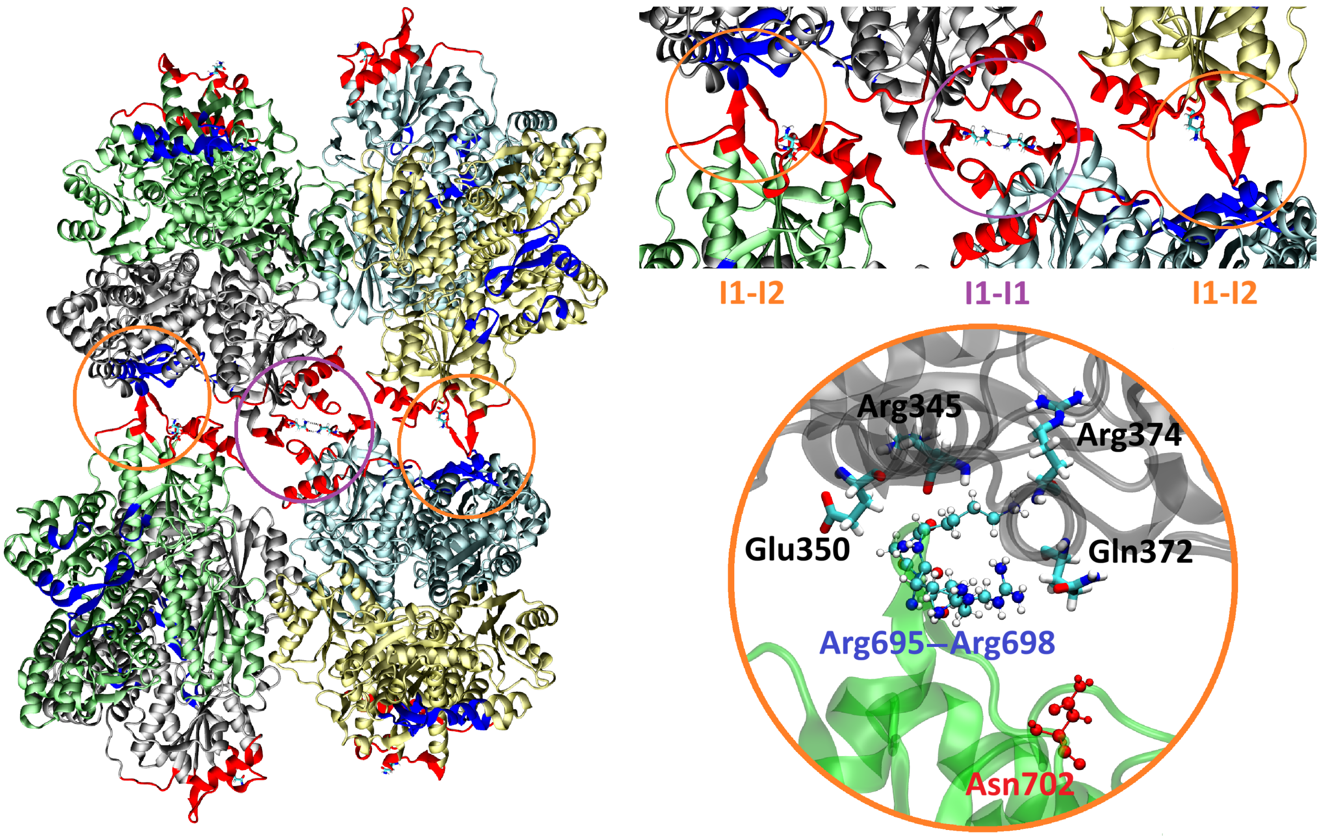

Figure S16: Detailed view of PFKL R-state filament cryoEM structure (PDB ID: 8W2I[8]). Each chain is colored differently. Interface 1 residues are highlighted in red and Interface 2 residues in blue. Asn702 residues are shown in licorice representation. The zoomed-in panel depicts key residues involved in the Interface 1–Interface 2 contacts, with Interface 2 residues shown in licorice and Interface 1 residues in CPK representation. The Interface 1–Interface 2 contacts primarily involve residues 695–698 from Interface 1 and residues Arg345, Glu350, Gln372, and Arg374 from Interface 2. Note that the distance between Asn702 in Interface 1 and Gln372 in Interface 2 is too large to permit hydrogen bond formation.

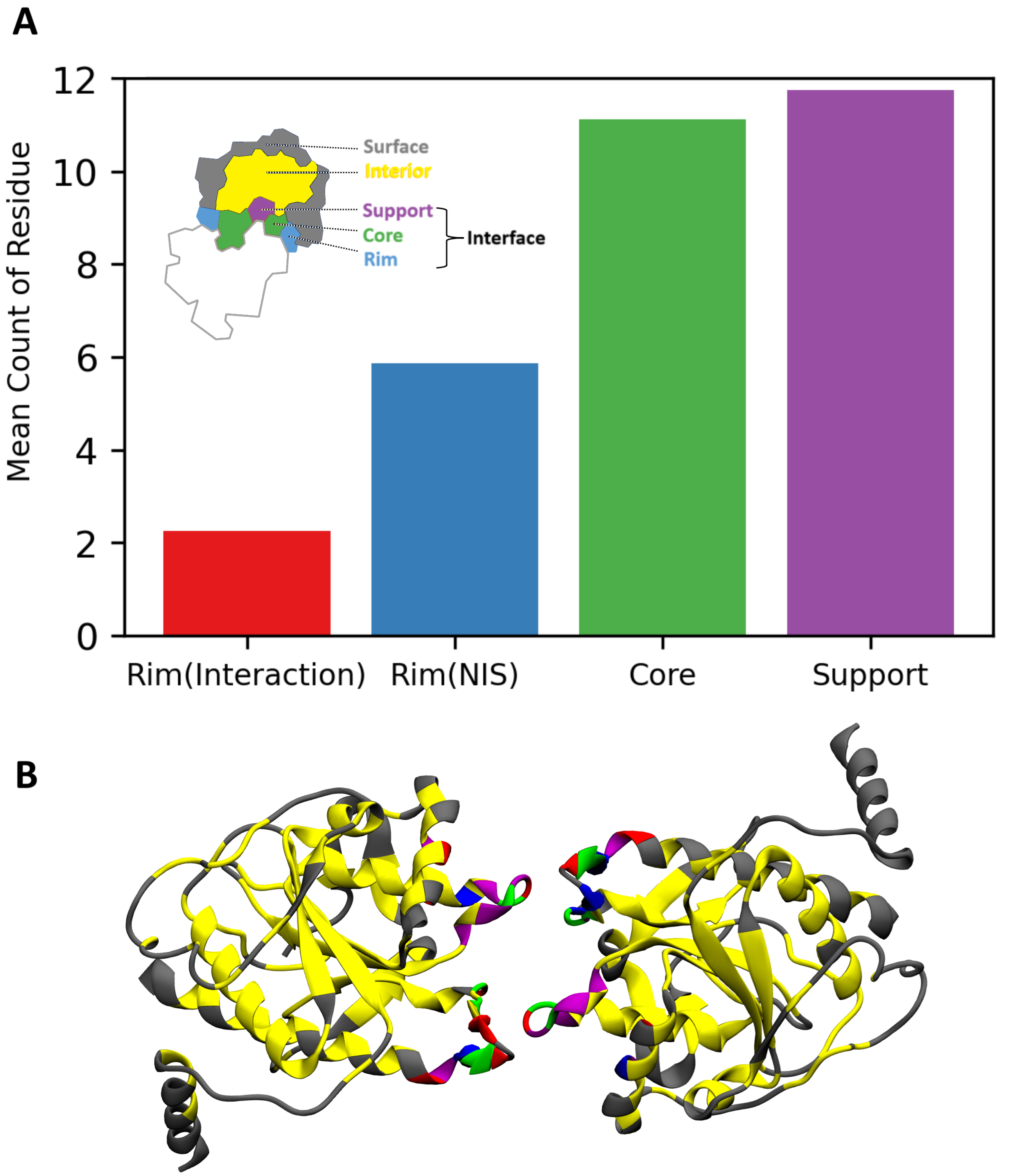

Figure S17: (A) The fragment interface regions obtained from a cluster structure computed from the 48 frames representing the three trajectories analyzed by the MICLOT tool.[26] NIS stands for non-interacting surface. Means from the region are identified using the theoretical MaxASA values from the paper by Tien et al.[9] The inset figure shows a representative cartoon of the protein regions. (B) The residues of PFKL fragments are colored based on the region they belong to.

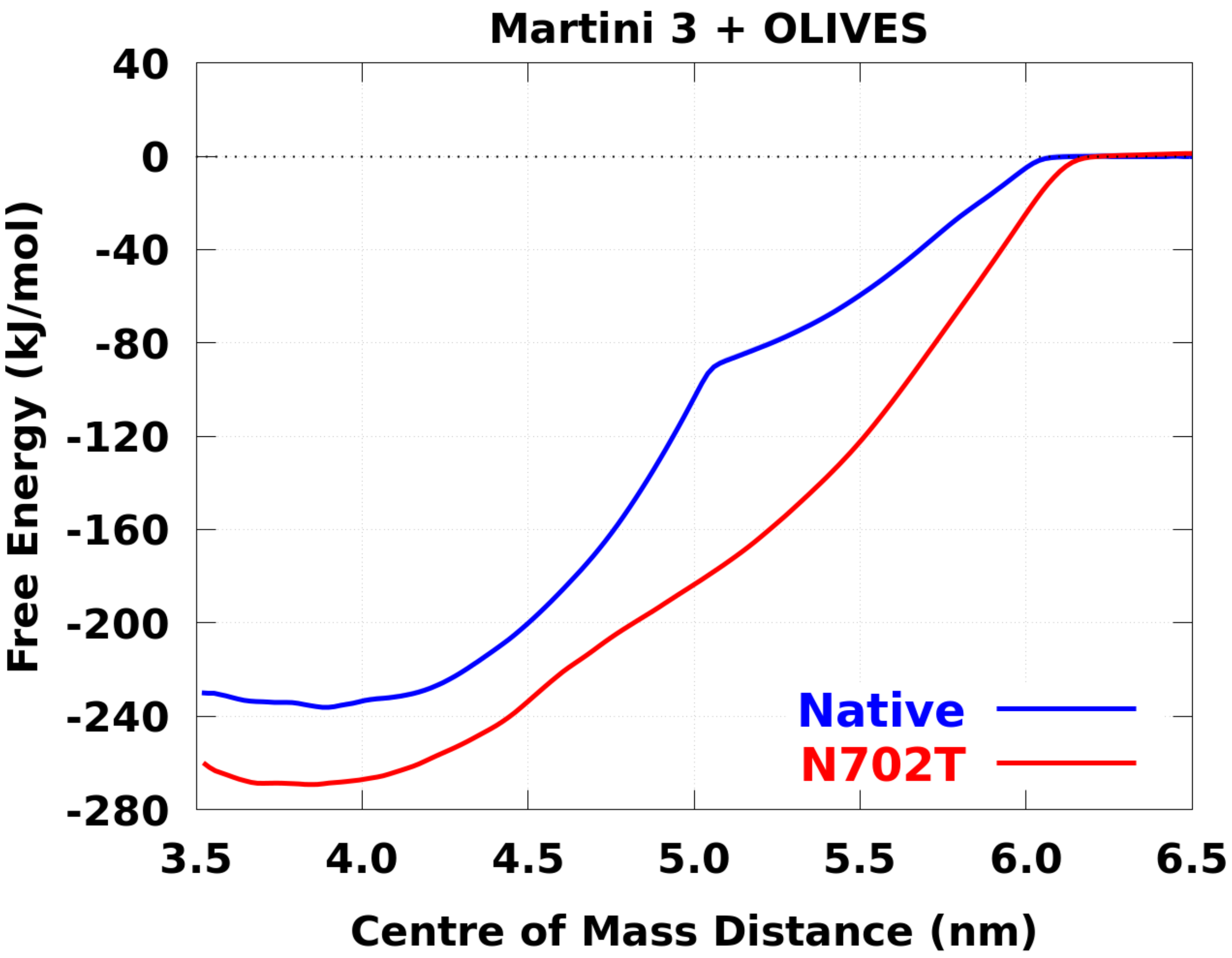

Figure S18: Free energy profiles of Interface 1–Interface 1 interaction for native PFKL and its N702T mutant (a PFKP-like mutation) obtained using Martini 3 with OLIVES model.

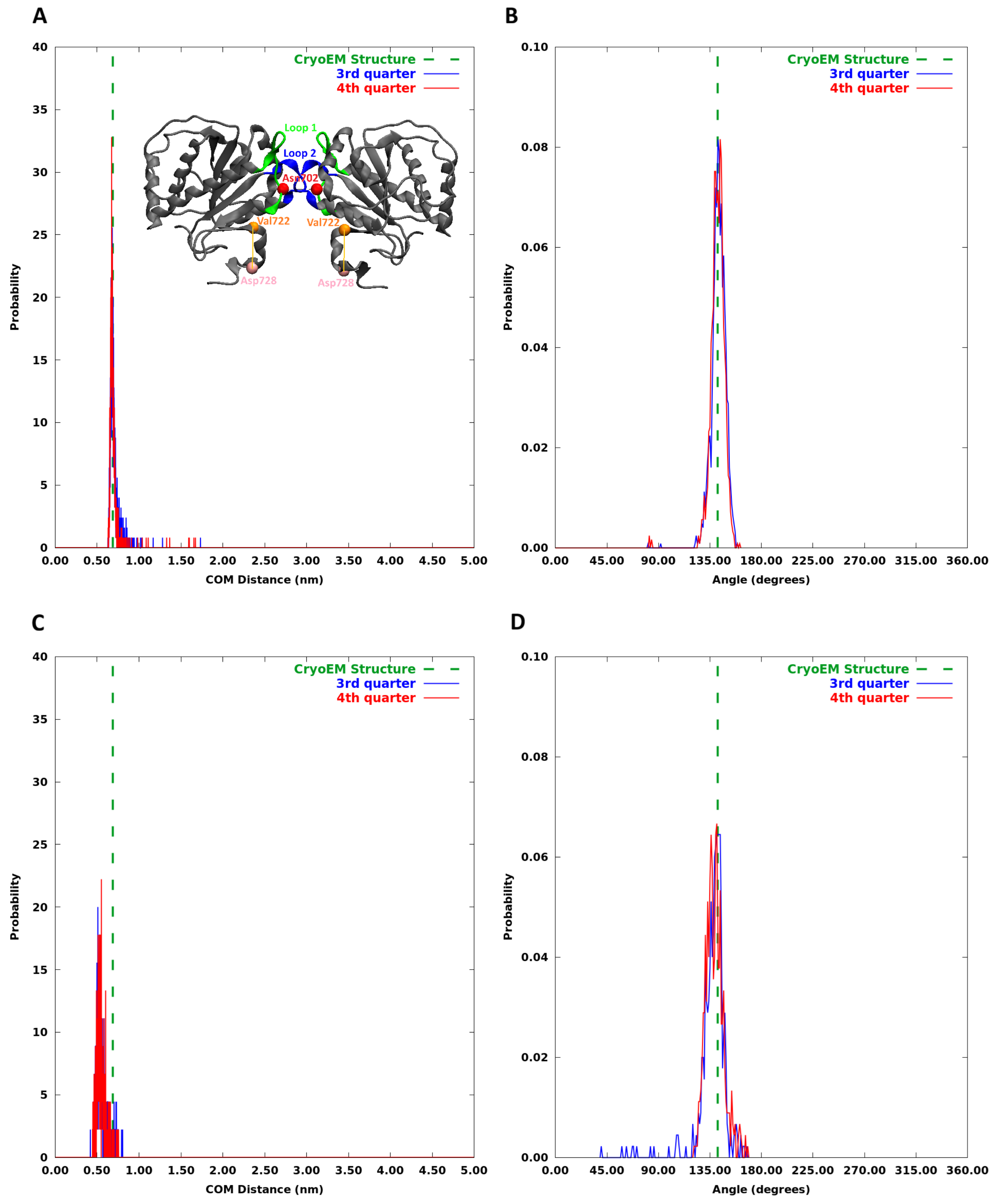

Figure S19: The histograms for (A) Asn702–Asn702 COM distance, and (B) Angle between vectors defined by Val722 and Asp728 C_α atoms for the native PFKL fragments in CHARMM36m force field. The histograms for (C) Asn702–Asn702 COM distance, and (D) Angle between vectors defined by Val722 and Asp728 backbone beads for the lowest minimum for the native PFKL fragments in Martini 3 force field with an extra hydrogen-bonding term. The dashed lines show the values for the CryoEM structure.

Transient Interactions and Filament Stability with Added Hydrogen-Bonding Terms in Martini 3 Force Field

To evaluate our modified Martini 3 force field, which includes an additional hydrogen bond for the side chains of Asn702 residues, we performed 10 μs unbiased simulations (three replicas) of two PFKL tetramers randomly placed in the simulation box. The same simulation procedure as in Martini 3, with randomly positioned tetramers, was used for the simulations of the modified Martini 3 force field with an extra hydrogen bond for Asn702 residue's side-chain. Although filament formation was not observed within the limited simulation timescale, the extra hydrogen-bonding term significantly strengthened the Interface 1–Interface 1 interactions (see Figure S20). Moreover, starting from a preformed filament yielded a stable filament over 15 μs of simulation (data not shown). These findings indicate that this minimal modification to the Martini 3 force field is sufficient to efficiently stabilize the Interface 1–Interface 1 interaction.

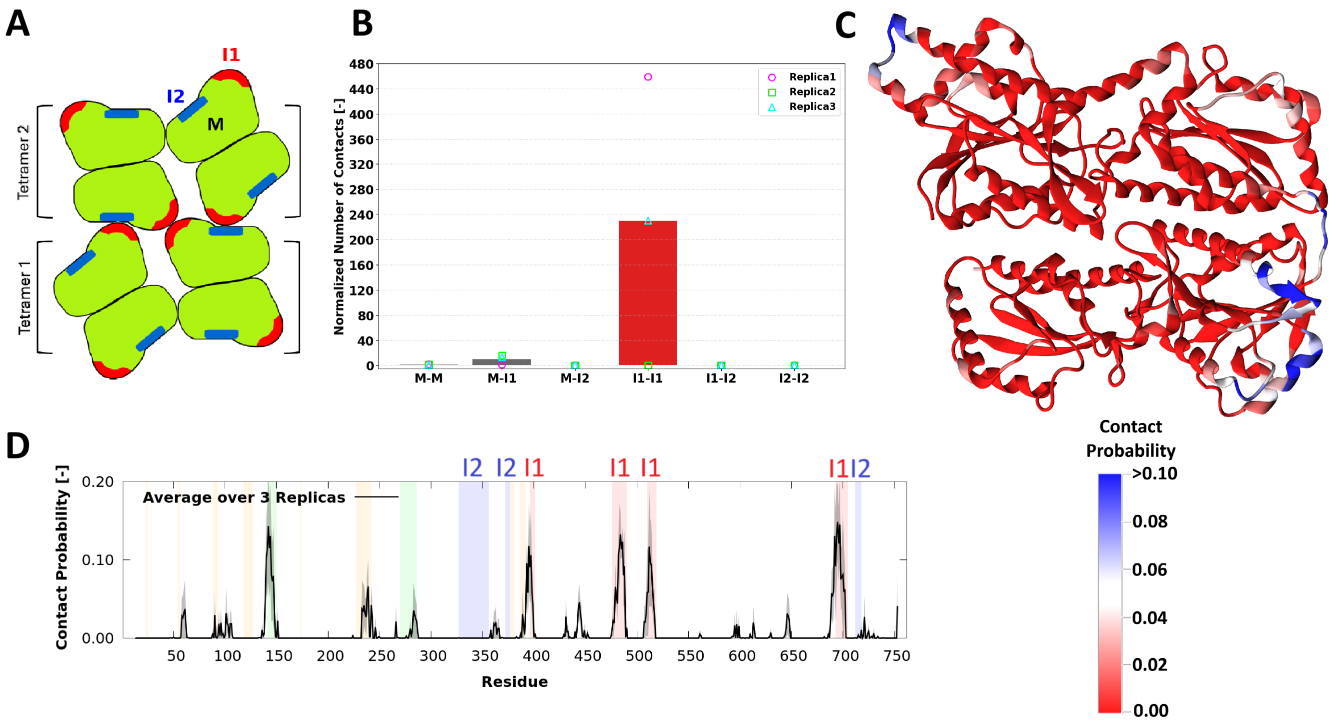

Figure S20: Interactions between PFKL tetramers in Martini 3 with added hydrogen-bonding term on Asn702 side-chain. (A) A representative cartoon of two PFK1 tetramers stacked in a filament. Individual monomers forming each tetramer are shown as a green surface. The red regions show Interface 1, while the blue regions indicate Interface 2 in each monomer. (B) Average number of contacts normalized by SASA values of two PFKL tetramers over 3 simulation replicas started from random initial positions. M indicates the four monomers in each tetramer, excluding the residues of Interfaces 1 and 2. The points indicate the value for each replica. (C) The residue contact probabilities for PFKL mapped on the structure of the monomer. (D) The average contact probability per residue for PFKL in simulations started from random initial positions, with the average taken over eight monomers and three replicas. Two beads were considered in contact if their distance was less than 0.7 nm. The shaded areas in gray show the standard error of the mean (SEM). The residue ranges of Interface 1 and Interface 2 are highlighted in red and blue, respectively. The shaded orange and green regions are the ATP binding sites and two important epitope regions, respectively.

Free Energies Convergence Analysis

We tested the convergence of the free energy profiles by using the first quarter, the second quarter, the third quarter, and the fourth quarter of the trajectories from each window. We also verified that the histograms had sufficient overlap for the free energy profiles reconstruction; see Figure S21-Figure S24. In Figure 5 and Figure 6, we plotted the free energy profiles for the second halves (the third and fourth quarters) of the trajectories. To verify that the native structure was properly sampled at close distances, we analyzed additional variables, including the distance between Asn702 residues COM, and the angle between the vectors defined by Val722 and Asp728 residues C_α atoms. Figure S19 shows the results for the minimum of the free energy profiles obtained from CHARMM36m force field and Martini 3 with an extra hydrogen-bonding term.

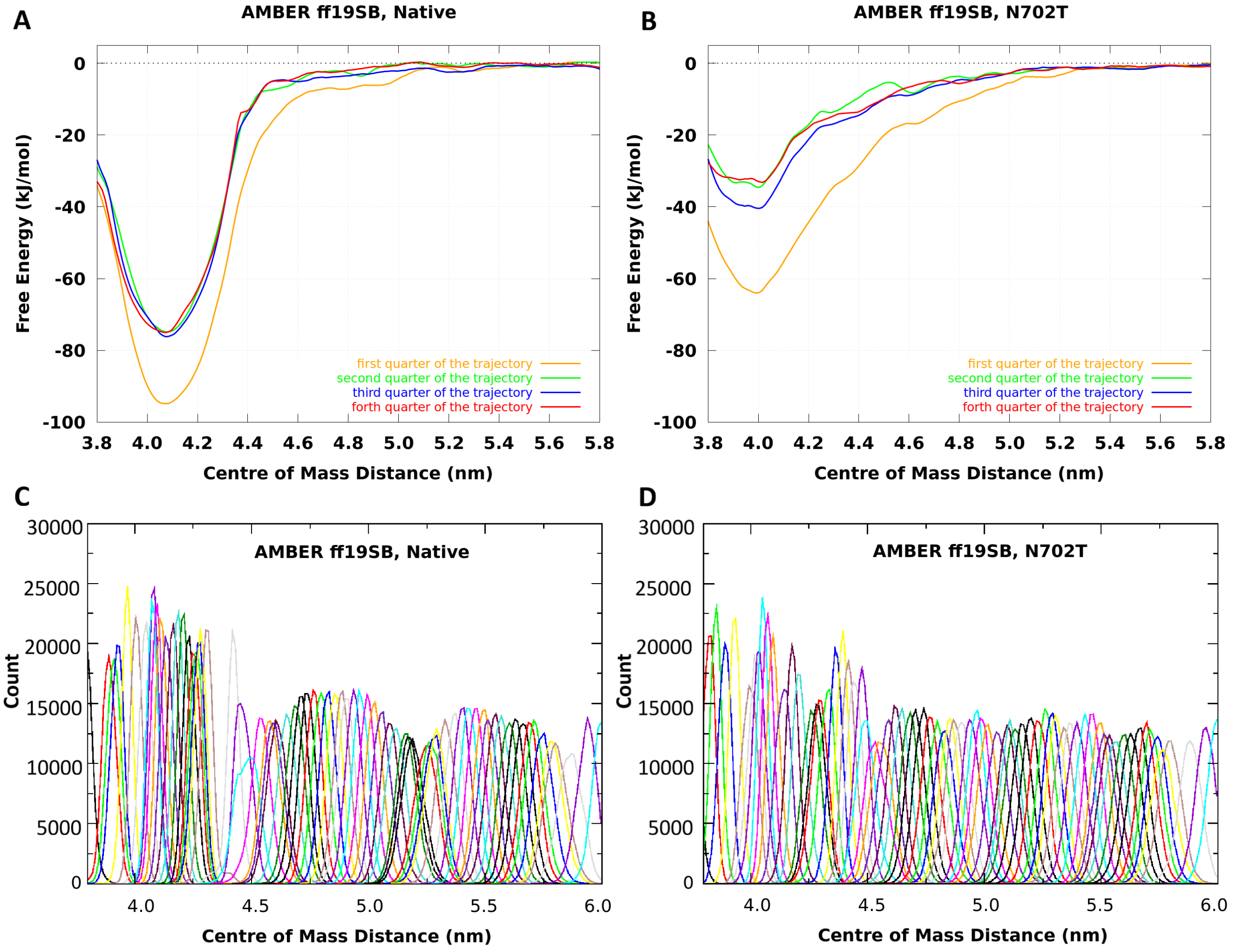

Figure S21: Free energy profiles obtained from the first, the second, the third, and the fourth quarter of the trajectories for (A) Native and (B) N702T mutant PFKL fragments in AMBER ff19SB force field. Umbrella histograms for (C) Native and (D) N702T mutant PFKL fragments.

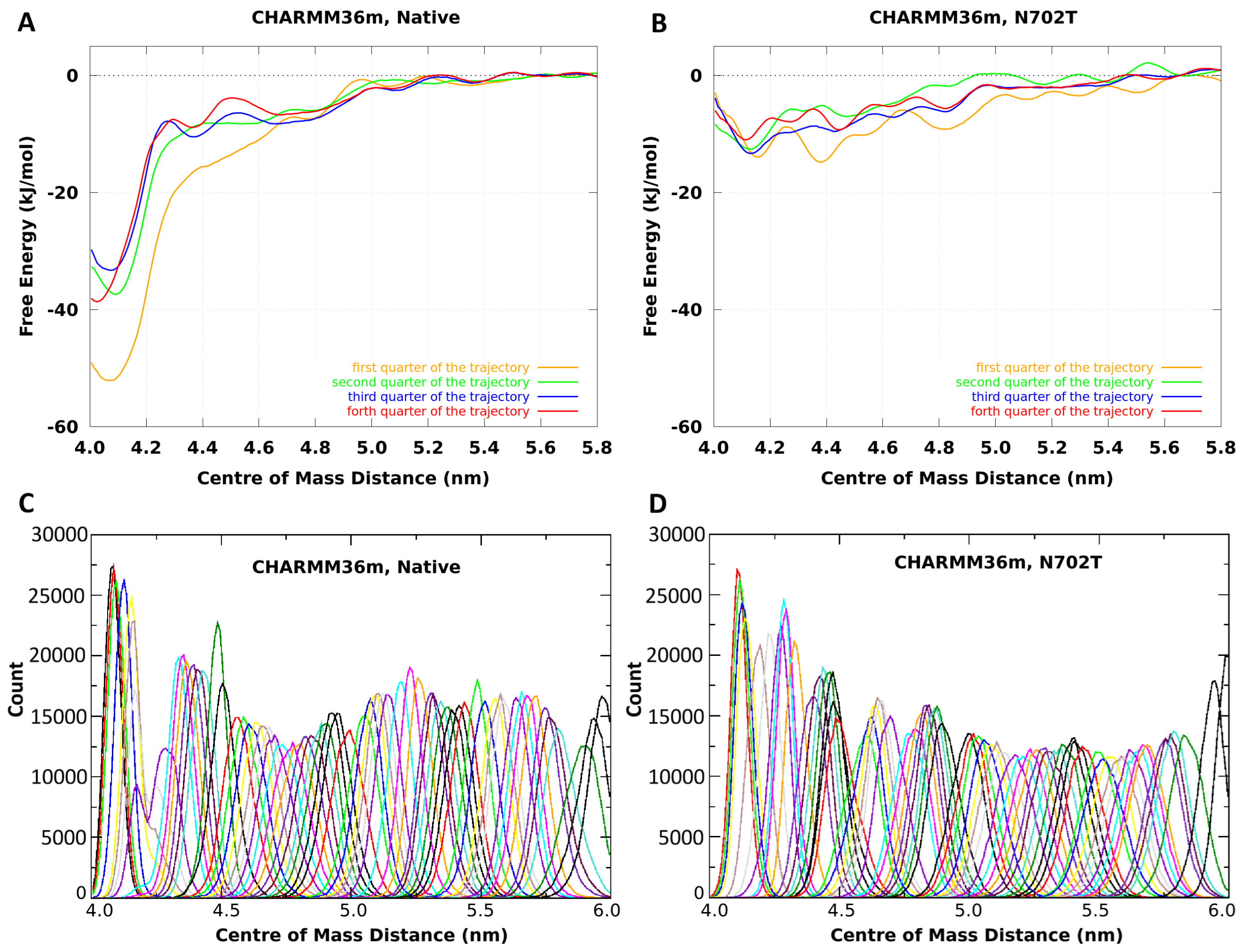

Figure S22: Free energy profiles obtained from the first, the second, the third, and the fourth quarter of the trajectories for (A) Native and (B) N702T mutant PFKL fragments in CHARMM36m force field. Umbrella histograms for (C) Native and (D) N702T mutant PFKL fragments.

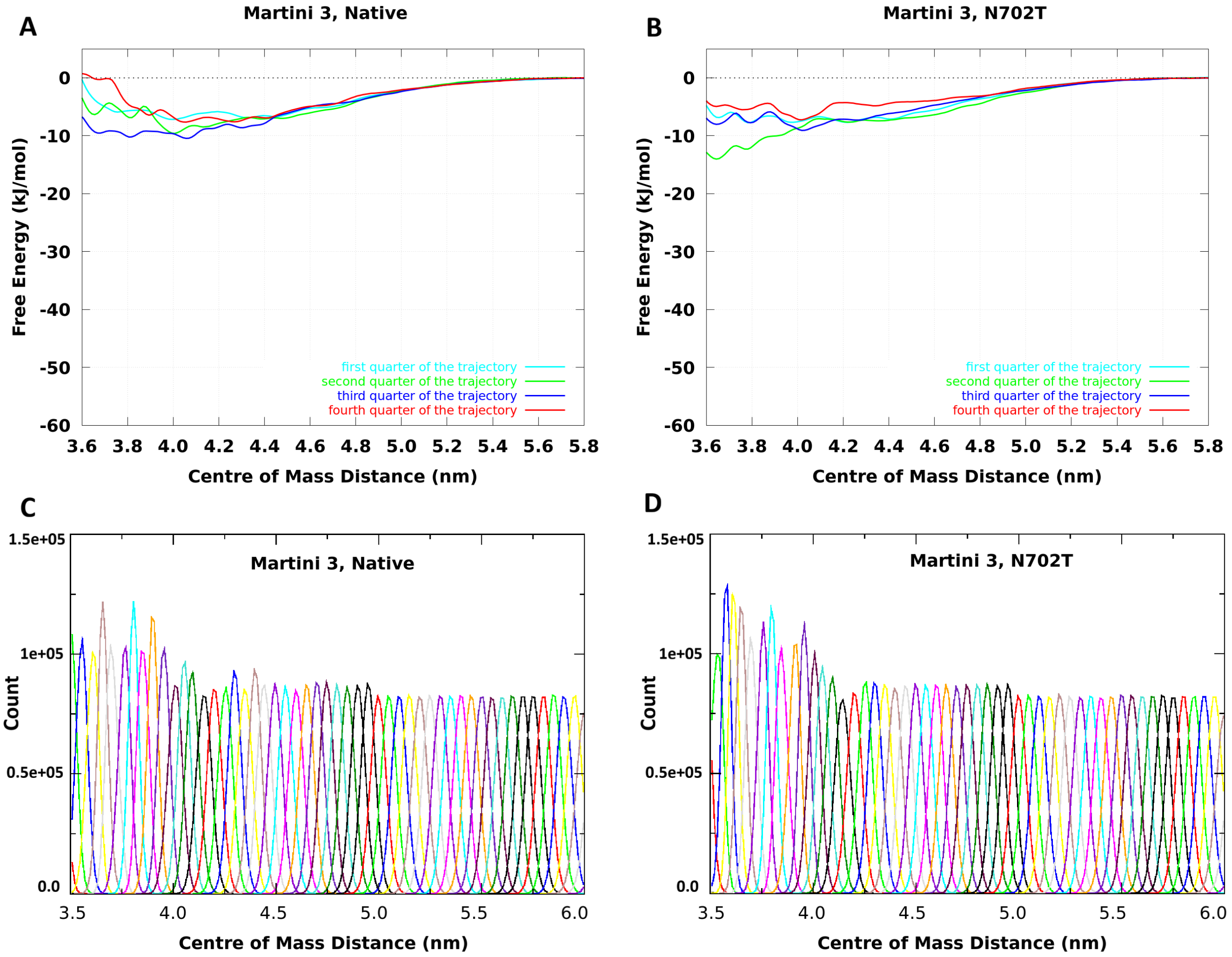

Figure S23: Free energy profiles obtained from the first, the second, the third, and the fourth quarter of the trajectories for (A) Native and (B) N702T mutant PFKL fragments in Martini 3 force field. Umbrella histograms for (C) Native and (D) N702T mutant PFKL fragments.

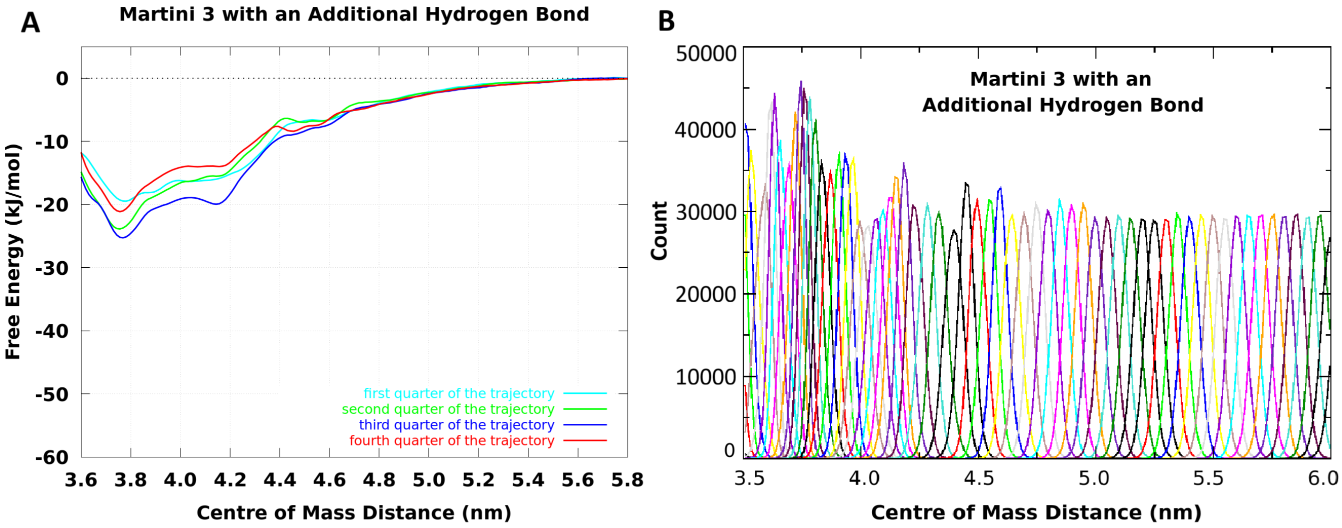

Figure S24: (A) Free energy profiles obtained from the first, the second, the third, and the fourth quarter of the trajectories, and (B) Umbrella histogram for the Native PFKL fragments in Martini 3 force field with an extra hydrogen bond.
